# Quantification of Anti-Aggregation Activity of Chaperones: A Test-System Based on Dithiothreitol-Induced Aggregation of Bovine Serum Albumin

**DOI:** 10.1371/journal.pone.0074367

**Published:** 2013-09-10

**Authors:** Vera A. Borzova, Kira A. Markossian, Dmitriy A. Kara, Natalia A. Chebotareva, Valentina F. Makeeva, Nikolay B. Poliansky, Konstantin O. Muranov, Boris I. Kurganov

**Affiliations:** 1 Department of Molecular Organization of Biological Structures, Bach Institute of Biochemistry, Russian Academy of Sciences, Moscow, Russia; 2 Department of Biochemistry, Faculty of Biology, Lomonosov Moscow State University, Moscow, Russia; 3 Department of Chemical and Biological Processes Kinetics, Emanuel Institute of Biochemical Physics, Russian Academy of Sciences, Moscow, Russia; Aligarh Muslim University, India

## Abstract

The methodology for quantification of the anti-aggregation activity of protein and chemical chaperones has been elaborated. The applicability of this methodology was demonstrated using a test-system based on dithiothreitol-induced aggregation of bovine serum albumin at 45°C as an example. Methods for calculating the initial rate of bovine serum albumin aggregation (*v*_agg_) have been discussed. The comparison of the dependences of *v*_agg_ on concentrations of intact and cross-linked α-crystallin allowed us to make a conclusion that a non-linear character of the dependence of *v*_agg_ on concentration of intact α-crystallin was due to the dynamic mobility of the quaternary structure of α-crystallin and polydispersity of the α-crystallin–target protein complexes. To characterize the anti-aggregation activity of the chemical chaperones (arginine, arginine ethyl ester, arginine amide and proline), the semi-saturation concentration [L]_0.5_ was used. Among the chemical chaperones studied, arginine ethyl ester and arginine amide reveal the highest anti-aggregation activity ([L]_0.5_ = 53 and 58 mM, respectively).

## Introduction

Folding of newly synthesized polypeptide chains can be accompanied by the formation of proteins prone to aggregation. Non-native proteins which implied to aggregation are also formed under stress conditions. Aggregation of non-native proteins may be prevented by small heat shock proteins (sHsps) and also by some low-molecular-weight compounds, so-called “chemical chaperones”.

sHsps, as a class of molecular chaperones, form a large family of ubiquitous proteins with molecular mass of subunit in the range 12–40 kDa, which are able to prevent protein aggregation. α-Crystallin is a representative of a family of sHsps, exhibits chaperone-like properties, including the ability to prevent the precipitation of denatured proteins [1]–[3]. The ability of α-crystallin to suppress heat-induced aggregation of proteins is a result of hydrophobic interactions with denatured proteins, and this ability increases when α-crystallin is heated [4], [5]. sHsps, including α-crystallin, form highly dynamic assemblies of different size and composition [6], [7]. Benesch and co-workers [6], [8], [9] suppose that the dynamic quaternary structure play an important role in sHsps chaperone function. There is some evidence that the dissociated forms of sHsps are the chaperone-active species which interact with target proteins and are subsequently sequestered into high mass complexes [10]–[13]. Ample evidence demonstrates the complexation of α-crystallin with nonnative proteins [1], [14]–[18]. The formation of complexes between dissociated forms of α-crystallin and target substrates, muscle glyceraldehyde 3-phosphate dehydrogenase (GAPDH) or glycogen phosphorylase *b* (Ph*b*), at elevated temperatures has been demonstrated in our studies [19]–[22].

The data on the importance of quaternary structure of α-crystallin for chaperone-like activity and the location of substrate-binding site(s) are contradictory. It was shown that subunit exchange was not required for chaperone function of α-crystallin. Bovine α-crystallin cross-linked with glutaraldehyde under conditions designed to minimize intermolecular reactions was able to inhibit the thermally-induced precipitation of β_L_-crystallin and appeared to be more effective than the native protein under the same conditions [23]. Horwitz et al. [24] have shown that native oligomeric state of α-crystallin may not be essential for its ability to suppress non-specific aggregation, since prepared tetramers of α-crystallin had the same chaperone-like activity as the native oligomeric α-crystallin. However, according to Sharma and Ortwerth [25], age-related cross-linking of α-crystallin reduces its chaperone-like activity.

Among chemical chaperones arginine (Arg) is the most effective additive in suppressing heat- and dithiothreitol (DTT)-induced aggregation of proteins [26]–[31] and protein aggregation during *in vitro* folding [32]. It is suggested that Arg does not facilitate refolding, but can suppress aggregation of the proteins during refolding [33], [34].

Solubility measurements of 20 amino acids and model peptides showed that a majority of amino acids chains, in particular aromatic amino acids, of proteins favorably interact with Arg. Such favorable interactions should be reflected on Arg binding to protein surfaces [35]–[38]. Tomita et al. [31] showed that heat-induced aggregation of lysozyme at around the isoelectric point occurred in a two-step process: formation of start aggregates, followed by further growth mediated by their sticking with diffusion-limited cluster-cluster aggregation. In the presence of Arg, the diffusion-limited regime changed to reaction-limited cluster-cluster aggregation. According to the data presented by Srinivas et al. [39], [40], Arg is able to affect the tertiary and quaternary structure of α-crystallin and enhances the dynamics of the subunit assembly leading to enhanced chaperone-like activity.

It is important that Arg derivatives such as arginine ethylester (ArgEE) and arginine amide (ArgAd) are more effective additives for both heat-induced and refolding-induced irreversible misfolding of lysozyme than Arg [27]–[29], [41].

Anti-aggregation activity of proline (Pro), one of the osmolytes behaving as a chemical chaperone, was demonstrated in *in vivo* and *in vitro* experiments [22], [42]–[45]. Pro is found to prevent aggregation during protein refolding [42], [43], [46], [47]. Experimental evidence suggests that Pro inhibits protein aggregation by binding to folding intermediate(s) and trapping the folding intermediate(s) into enzymatically inactive, “aggregation-insensitive” state(s) [48], [49]. As shown by Eronina et al. [50], the suppression of aggregation at high Pro concentrations (>0.3 M) was mainly due to the protective action of Pro on the stage of unfolding of the Ph*b* molecule.

Main problems facing the biochemists studying the anti-aggregation functions of molecular chaperones are the following: how molecular chaperones realize their anti-aggregation activity, how to compare the anti-aggregation activities of molecular chaperones of different classes and how to quantitatively characterize the mutual effects of molecular chaperones of different classes. To solve these problems, the investigator should have the strict quantitative methods of the estimation of the anti-aggregation activity of chaperones at his disposal. The goal of the present work is to elaborate the theoretical approaches to quantification of the anti-aggregation activity of chaperones and to demonstrate the applicability of these approaches using a new test-system based on DTT-induced aggregation of bovine serum albumin (BSA).

BSA is a water-soluble monomeric protein with molecular mass of 66.4 kDa [51] and isoelectric point around 4.7–5.2 [52]. Polypeptide chain of BSA consists of 583 amino acid residues [53]. The three-dimensional structure of BSA is composed of three homologous domains (I, II, III), each formed by six helices [54]. Tertiary structure is well defined: 17 disulphide bonds give some rigidity of each sub-domain but allow significant modification in the shape and size of the protein under different external conditions [51], [55], [56]. At neutral pH the disulphide bridges are buried in the protein molecule and not exposed to the solvent [57]. Besides, a unique free cystein (Cys-34) is located in domain I in a hydrophobic pocket of the BSA molecule [58]. BSA has two tryptophans (Trp), embedded in two different domains: Trp-134, located in proximity of the protein surface, but buried in a hydrophobic pocket of domain I, and Trp-214, located in an internal part of domain II [59].

The treatment of BSA molecules with DTT reduces S-S into SH [60]. As a result, the α-helical structure is disrupted and the β-structure is formed after unfolding, coupled with reducing disulfide bonds of BSA [61], [62]. None of the disulphide bonds in BSA molecule is accessible to reducing agents in the pH range 5–7, however, between pH 7 and 10 approximately five disulfide bonds became available for reduction [57]. When the temperature increases from 35 to 55°C, the reduction of disulfide bonds also increases [63].

Sogami at al. [64] showed that BSA was prone to intramolecular disulfide-interchange reactions which markedly broaden the population of the protein molecules. The structural fluctuations of BSA are internal without significant effect on the external shape of the protein molecules. It is supposed that fluctuations in disulfide pairing are responsible for the microheterogeneity of BSA [64].

Gobbo et al. [65] proposed a test-system based on DTT-induced aggregation of BSA for the analysis of the anti-aggregation activity of sHsp27. BSA aggregation kinetics (50 mM Na-phosphate buffer, pH 7) at 45 °C was monitored spectrophotometrically at 340 nm. This chaperone quantification test was based on the capacity of Hsp 27 to suppress DTT-induced aggregation of BSA. However, the authors did not represent the kinetic curves of aggregation in the absence and in the presence of Hsp27 and did not discuss the quantitative methods of estimation of the anti-aggregation activity of the chaperone. Therefore it is difficult to use the work by Gobbo et al. [65] in practice.

In the present work we studied the kinetics of DTT-induced aggregation of BSA at various concentrations of the protein and DTT using dynamic light scattering (DLS). It has been demonstrated that a test-system based on the DTT-induced aggregation of BSA may be used for the quantitative estimation of the ability of different agents to suppress protein aggregation. In particular, the chaperone-like activities of intact and cross-linked α-crystallin, a representative of the family of Hsps, and of chemical chaperones Arg, ArgEE, ArgAd and Pro were quantified.

## Materials and Methods

### Chemicals

BSA (catalogue no. A7638, 99+% of purity), DL-dithiothreitol (99% of purity), L-arginine monohydrochloride (Arg), L-arginine ethylester (ArgEE), L-arginine amide (ArgAd) and L-proline (Pro) (reagent grade) were purchased from Sigma-Aldrich and used without further purification.

### Sample Preparation

All solutions for the experiments were prepared using deionized water obtained with Easy-Pure II RF system (Barnstead, USA). BSA samples were prepared by dissolving solid BSA in 0.1 M phosphate buffer solutions at pH 7.0. BSA concentration was determined spectrophotometrically at 280 nm using the absorption coefficient 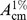 of 6.58 [66].

### Isolation of α-Crystallin

α-Crystallin was isolated from freshly excised eye lenses of 2-year-old steers (*Bos taurus*). The eye lenses were obtained from a local slaughter-house “Pushkinskii Myasnoi Dvor”, located at Sokolovskaya St. 1, Pushkino, Moscow Region, Russia. Authors confirm that they have permission from the slaughterhouse to use these animal parts. Purification of α-crystallin, was performed according to the procedure described earlier [67], [68]. α-Crystallin concentration was determined spectrophotometrically at 280 nm using the absorption coefficient 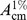 of 8.5 [5].

### Preparation of Cross-Linked α-Crystallin

Cross-linking of α-crystallin was performed according to Augusteyn [69] with some modification. The intact protein (0.03 mM) was incubated in 40 mM phosphate buffer (pH 7.0), containing 150 mM NaCl, 1 mM EDTA and 3 mM NaN_3_, with 3 mM glutaraldehyde at 20°C for 30 h. 3 mM DTT was added to block any non-reactive aldehyde groups and then the protein was dialyzed against the same buffer. The obtained samples were centrifuged at 4500 g for 30 min, using MiniSpin+, Eppendorf centrifuge and the supernatant was passed through a size-exclusion chromatography (SEC) column. The concentration of cross-linked α-crystallin was determined by micro-biuret method [70].

### Size-Exclusion Chromatography

The protein samples were loaded onto the column (Toyopearl TSK-gel HW-55 fine; 2.5 cm × 90 cm) and separated into the fractions at a flow rate of 1.7 ml/min (20°C). The column was pre-calibrated with the following proteins from (Sigma-Aldrich): thyroglobulin (660 kDa), catalase (440 kDa), aldolase (158 kDa), BSA (67 kDa), α-crystallin (20 kDa). The relative error for protein mass determination was 4%.

### Sodium Dodecyl Sulfate-Polyacrylamide Gel Electrophoresis (SDS−PAGE)

The polypeptide composition of the protein samples was analyzed by electrophoresis in 15% PAAG in the presence of SDS and DTT [71]. Sigma-Aldrich proteins α-lactalbumin (14.2 kDa), trypsin inhibitor (20.1 kDa), carbonic anhydrase (29 kDa), ovalbumin (45 kDa) and BSA (66 kDa) were used as standards. The gels were stained with Coomassie R-250 and scanned with an Epson Perfection 4180 photoscanner. The images were analyzed with ImageJ 1.41n program.

### Determination of Refractive Index, Density and Dynamic Viscosity

The values of the refractive index of Arg, ArgEE, ArgAd and Pro solutions at the different concentrations (0.1 M Na-phosphate buffer, pH 7.0) were determined in ABBEMAT 500 refractometer (Anton Paar, Austria) at 45°C. Density of Arg, ArgEE, ArgAd and Pro solutions were determined in density meter DMA 4500 (Anton Paar, Austria). Dynamic viscosities of the solutions were determined in automatic microviscosimeter (Anton Paar, Austria) in system 1.6/1.500 mm at 45°C. The obtained values of the refractive index, density and dynamic viscosity of Arg, ArgEE, ArgAd and Pro solutions are given in Table 1. The values of refractive index and dynamic viscosity of Arg, ArgEE, ArgAd and Pro solutions were used in the DLS measurements.

**Table 1:** The values of refractive index (*n*), density (ρ) and dynamic viscosity (η) of solutions of arginine, arginine ethylester, arginine amide and proline at 45°C (0.1 M Na-phosphate buffer, pH 7.0).

Concentration (mM)	*n*	ρ (g/cm^3^)	η (mPa⋅s)
	**Arginine**		
0	1.3320±0.0002	0.99070±0.00005	0.4214±0.0003
50	1.3340±0.0002	0.99870±0.00005	0.4339±0.0003
75	1.3351±0.0002	0.99995±0.00005	0.4398±0.0002
100	1.3361±0.0002	1.00120±0.00005	0.4461±0.0002
150	1.3380±0.0002	1.00370±0.00005	0.4574±0.0003
200	1.3402±0.0002	1.00620±0.00005	0.4613±0.0002
400	1.3486±0.0002	1.01620±0.00005	0.5160±0.0002
	**Arginine ethylester**		
0	1.3328±0.0002	1.00689±0.00005	0.6372±0.0003
25	1.3342±0.0002	1.00781±0.00005	0.6463±0.0003
50	1.3365±0.0002	1.00962±0.00005	0.6690±0.0002
75	1.3370±0.0002	1.01193±0.00005	0.6702±0.0002
100	1.3385±0.0002	1.01274±0.00005	0.6826±0.0003
150	1.3410±0.0002	1.01588±0.00005	0.7022±0.0002
	**Arginine amide**		
0	1.3328±0.0002	1.00532±0.00005	0.6358±0.0003
10	1.3325±0.0002	1.00694±0.00005	0.6377±0.0003
30	1.3324±0.0002	1.00909±0.00005	0.6443±0.0002
50	1.3335±0.0002	1.01104±0.00005	0.6501±0.0002
75	1.3349±0.0002	1.01305±0.00005	0.6582±0.0003
100	1.3359±0.0002	1.01489±0.00005	0.6646±0.0002
150	1.3382±0.0002	1.01758±0.00005	0.6787±0.0002
	**Proline**		
0	1.3327±0.0002	1.00602±0.00005	0.6282±0.0003
150	1.3361±0.0002	1.01093±0.00005	0.6563±0.0003
300	1.3398±0.0002	1.01700±0.00005	0.7010±0.0002
500	1.3440±0.0002	1.02364±0.00005	0.7420±0.0002
750	1.3483±0.0002	1.02972±0.00005	0.7842±0.0003
1000	1.3525±0.0002	1.03777±0.00005	0.8456±0.0002

### Light Scattering Intensity Measurements

For light scattering measurements a commercial instrument Photocor Complex (Photocor Instruments, Inc., USA) was used. A He-Ne laser (Coherent, USA, Model 31-2082, 632.8 nm, 10 mW) was used as a light source. DynaLS software (Alango, Israel) was used for polydisperse analysis of DLS data. The diffusion coefficient *D* of the particles is directly related to the decay rate τ_c_ of the time-dependent correlation function for the light scattering intensity fluctuations:
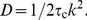
(1)


In this equation *k* is the wave number of the scattered light, *k* = (4π*n*/λ)sin(θ/2), where *n* is the refractive index of the solvent, λ is the wavelength of the incident light in vacuum and θ is the scattering angle. The mean hydrodynamic radius of the particles, *R*_h_, can then be calculated according to Stokes-Einstein equation:
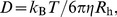
(2)where *k*_B_ is Boltzmann’s constant, *T* is the absolute temperature and η is the dynamic viscosity of the solvent.

The kinetics of DTT-induced aggregation of BSA was studied in 0.1 M Na-phosphate buffer, pH 7.0. The buffer was placed in a cylindrical cell with the internal diameter of 6.3 mm and preincubated for 5 min at a given temperature (45°C). Cells with stopper were used to avoid evaporation. The aggregation process was initiated by the addition of an aliquot of DTT to a BSA sample to the final volume of 0.5 ml. To study the effect of α-crystallin or Arg, ArgEE, ArgAd and Pro on BSA aggregation, the agents were added before the addition of DTT to a preheated solution of BSA. When studying the kinetics of aggregation of BSA, the scattering light was collected at a 90° scattering angle.

### Asymmetric Flow Field Flow Fractionation (A4F) with On-Line Multi-Angle Light Scattering (MALS), Ultraviolet (UV) and Refractive Index (RI) Detectors

The Eclipse 3 separation system (Wyatt Technology Corporation, USA) based on an Agilent HPLC pump (Agilent Technologies, USA) was used for A4F experiments. BSA sample or the mixture of BSA with cross-linked α-crystallin in 0.1 M Na-phosphate buffer, pH 7.0, preheated with 0.2 mM DTT for 2 h and cooled to room temperature 23°C was injected in the separation channel by Agilent autoinjection system (Agilent Technologies, USA). A 21.4 cm channel with a 350-mm channel spacer and ultrafiltration membrane made of regenerated cellulose with a 10-kDa molecular weight cut off (Wyatt Technology Corporation, USA) were used. The flow system was sequentially connected to UV detector (Agilent Technologies, USA), MALS detector (DAWN HELEOS II, Wyatt Technology Corporation, USA) and RI detector (Optilab T-rEX, Wyatt Technology Corporation, USA). The elution was performed with 0.1 M phosphate buffer (pH 7.0) at a flow rate at the channel outlet of 1 ml/min, 3 ml/min cross flow. The data from the detectors were processed in ASTRA software, version 5.3.4 (Wyatt Technology Corporation, USA) to yield the final profiles. The experiment was carried out at room temperature (23°C).

### Analytical Ultracentrifugation

Sedimentation velocity experiments were carried out at 45°C in a Model E analytical ultracentrifuge (Beckman), equipped with absorbance optics, a photoelectric scanner, a monochromator and an on-line computer. A four-hole An-F Ti rotor and 12 mm double sector cells were used. The rotor was preheated at 45°C in the thermostat overnight before the run. The sedimentation profiles of BSA, α-crystallin and their mixtures (0.1 M Na-phosphate buffer, pH 7.0 containing 10 mM NaCl; 2 mM DTT) were recorded by measuring the absorbance at 285 nm. All cells were scanned simultaneously against the buffer containing the same additives. The time interval between scans was 3 min. The sedimentation coefficients were estimated from the differential sedimentation coefficient distribution [*c*(*s*) versus *s*] or [*c*(*s*,*f*/*f*_0_) versus *s*] which were analyzed using SEDFIT program [72], [73]. The *c*(*s*) analysis was performed with regularization at a confidence level of 0.68 and a floating frictional ratio. The sedimentation coefficients were corrected to the standard conditions (a solvent with the density and viscosity of water at 20°C) using SEDFIT and SEDNTERP [74] programs.

### Calculations

OriginPro 8.0 SR0 software (OriginLab Corporation, USA) and Scientist (MicroMath, Inc., USA) software were for the calculations. To characterize the degree of agreement between the experimental data and calculated values, we used the coefficient of determination *R* ^2^ (without considering the statistical weight of the measurement results) [75].

## Theory. Quantification of the Chaperone-Like Activity

### Determination of the Initial Rate of Protein Aggregation

To characterize the anti-aggregation activity of a chaperone, we should measure the initial rate of aggregation of a model target protein and compare this rate with the corresponding value measured in the absence of a chaperone. Protein aggregates possess higher light scattering capability in comparison with the non*-*aggregated protein molecules. Therefore the simplest way to measure the initial rate of aggregation is registration of the increment of the light scattering intensity (*I*) or apparent optical absorbance (*A*). In the early stages, the acceleration of the aggregation process takes place, suggesting that aggregation proceeds through the nucleation stage. To characterize the initial rate of aggregation, the quadratic dependence on time (*t*) was proposed for the description of the initial parts of the kinetic curves of aggregation [76]:
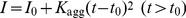
(3)or
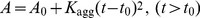
(4)where I_0_ and A_0_ are the initial value of the light scattering intensity and apparent optical absorbance, respectively, at t = 0 and t_0_ is the duration of lag period on the kinetic curve (t_0_ is a point in time at which the light scattering intensity or apparent optical absorbance begins to increase). Parameter k_agg_ is a measure of the initial rate of aggregation. Theoretical analysis shows that the quadratic law should be valid for nucleation-dependent aggregation [76], [77].

The applicability of Eq. (3) for the description of the initial parts of the kinetic curves of protein aggregation was demonstrated for thermal denaturation of Ph*b* [50], [76], [78], [79], GAPDH [80]–[82] and creatine kinase (CK) [83]from rabbit skeletal muscles and DTT-induced aggregation of α-lactalbumin [18] and insulin [84]. The practical significance of Eqs. (3) and (4) is as follows. First, the addition of a chaperone usually results in the elongation of the lag period on the kinetic curves, and the use of Eqs. (3) and (4) allows reliable determination of the duration of the lag period. It should be noted that the visual determination of the duration of the lag period on the kinetic curves is practically impossible. Second, the determination of parameter *k*_agg_ gives us a possibility to characterize quantitatively the anti-aggregation activity of the chaperone.

Consider different modifications of Eqs. (3) and (4). First, we can extend the time interval applicable for calculation of parameters *t*_0_ and *K*_agg_, if modify these equations as follows:
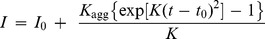
(5)or
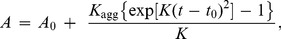
(6)where K is a constant which allows for the deviation from the quadratic dependence. It is significant that at t → t_0_ Eqs. (5) and (6) are transformed into Eqs. (3) and (4), respectively.

Secondly, one should bear in mind that in some cases the initial decrease in the light scattering intensity (or apparent optical absorbance) is observed on the kinetic curves of aggregation of a target protein, registered in the presence of chaperone, namely α-crystallin. Such a kinetic behavior was demonstrated, for example, when studying thermal aggregation of citrate synthase at 43°C [85] and β-amyloid peptide at 60°C [86]. There is a simple explanation for an unusual character of the kinetic curves of aggregation. Elevated temperatures induce dissociation of α-crystallin particles and a decrease in the light scattering intensity. This conclusion is substantiated by the data represented in our works [19], [20], [87], [88]. When a decrease in the light scattering intensity occurs in the initial part of the kinetic curves of aggregation, a reliable determination of the initial value of the light scattering intensity (*I*_0_) of the initial value of the apparent optical absorbance (*A*_0_) becomes impossible, and we can no longer apply Eq. (3) or Eq. (4). The differential forms of Eqs. (3) and (4) are useful in this case:
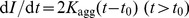
(7)or



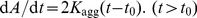
(8)Examples of using Eqs. (5) and (7) are given in the experimental part of the present work.

Analysis of the dependence of the initial rate of aggregation on the initial concentration of the target protein, [P]_0_, allows us to determine the order of aggregation with respect to the protein and draw inference about the rate-limiting stage of the aggregation process. The order of aggregation with respect to the protein (*n*) is calculated in accordance with the following equation:
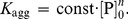
(9)


Below we will demonstrate that the knowledge of the *n* value is important for characterization of the anti-aggregation activity of chaperones of a protein nature.

In the case of thermal aggregation of Ph*b* (53°C; pH 6.8) [76] and GAPDH (45°C; pH 7.5) [82] the dependence of parameter *k*_agg_ on the initial concentration of the target protein is linear (*n* = 1). The kinetics of thermal aggregation of bovine liver glutamate dehydrogenase (GDH) at various concentrations of the protein was studied by Sabbaghian et al. [89] (50°C; pH 8.0). According to our calculations, the order of aggregation with respect to the protein calculated on the basis of these kinetic data is close to unity: *n* = 0.86±0.1. The case in which *n* = 1 means that unfolding of a protein molecule proceeds with a substantially lower rate than the following stages of aggregation of the unfolded protein molecules.

When unfolding of the protein molecule is a relatively fast process and the stages of aggregation become rate limiting, parameter *n* exceeds unity. For example, the analysis of the data on thermal aggregation of β_L_-crystallin from bovine lens at 60°C (pH 6.8) [68] and thermal aggregation of yeast alcohol dehydrogenase at 56°C (pH 7.4) [90] shows that parameter *n* is close to 2. An analogous situation was observed for aggregation of UV-irradiated GAPDH (37°C; pH 7.5; *n* = 2.1±0.2) [82].

It is of interest that the equation equivalent to Eq. (3) can be used for the description of the initial parts of the kinetic curves of aggregation in the experiments where temperature was elevated with a constant rate [91]:
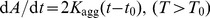
(10)where *T*_0_ is the initial temperature of aggregation, i.e., the temperature at which the light scattering intensity begins to increase, and *k*_agg_ is a parameter which characterizes the rate of aggregation. Parameters *T*_0_ and *k*_agg_ can be used for quantitative characterization of the ability of various agents to suppress protein aggregation. The applicability of Eq. (10) was demonstrated for aggregation of Ph*b*, GAPDH, CK and GDH.

According to theoretical views developed by Kurganov and co-workers [68], [91]–[93], the point in time *t* = *t*_0_ or point in temperature *T* = *T*_0_ corresponds to the appearance of start aggregates. A start aggregate contains hundreds of denatured protein molecules. The formation of the start aggregates proceeds on the all-or-none principle. The intermediate states between the non-aggregated protein and start aggregates are not detected in the system.

For completeness sake additional methods of determination of the initial rate of aggregation should be discussed. When analyzing the shape of the kinetic curves of aggregation of Ph*b* denatured by UV radiation [22], we observed that Eq. (3) is not fulfilled and, to characterize the initial rate of aggregation, we proposed to use the time interval (*t*_2I_) over which the initial value of the light scattering intensity is doubled. To calculate the *t*_2I_ value, the initial part of the dependence of the light scattering intensity on time was described by the stretched exponent:
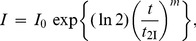
(11)where *m* is a constant. The reciprocal value of *t*_2I_, namely 1/*t*_2I_, may be considered as a measure of the initial rate of aggregation. The higher the 1/*t*_2I_ value, the higher is the initial rate of aggregation.

### Characterization of Anti-Aggregation Activity of Protein Chaperones

When analyzing the dependence of the initial rate of aggregation (*v*) on the concentration of protein chaperone, one should take into account two circumstances. First, the binding of a chaperone to a target protein is rather firm. The dissociation constant values for the chaperone–target protein complexes are of the order of magnitude of several nmoles per liter (see, for example, [94]). Suppression of aggregation is usually studied under the conditions where the initial concentrations of a chaperone and target protein exceed sufficiently the dissociation constant for the chaperone–target protein complex. This means that the dependence of *v* on [chaperone] is a titration curve which gives, in certain cases, information on the stoichiometry of the chaperone–target protein complex.

**Figure 1:**
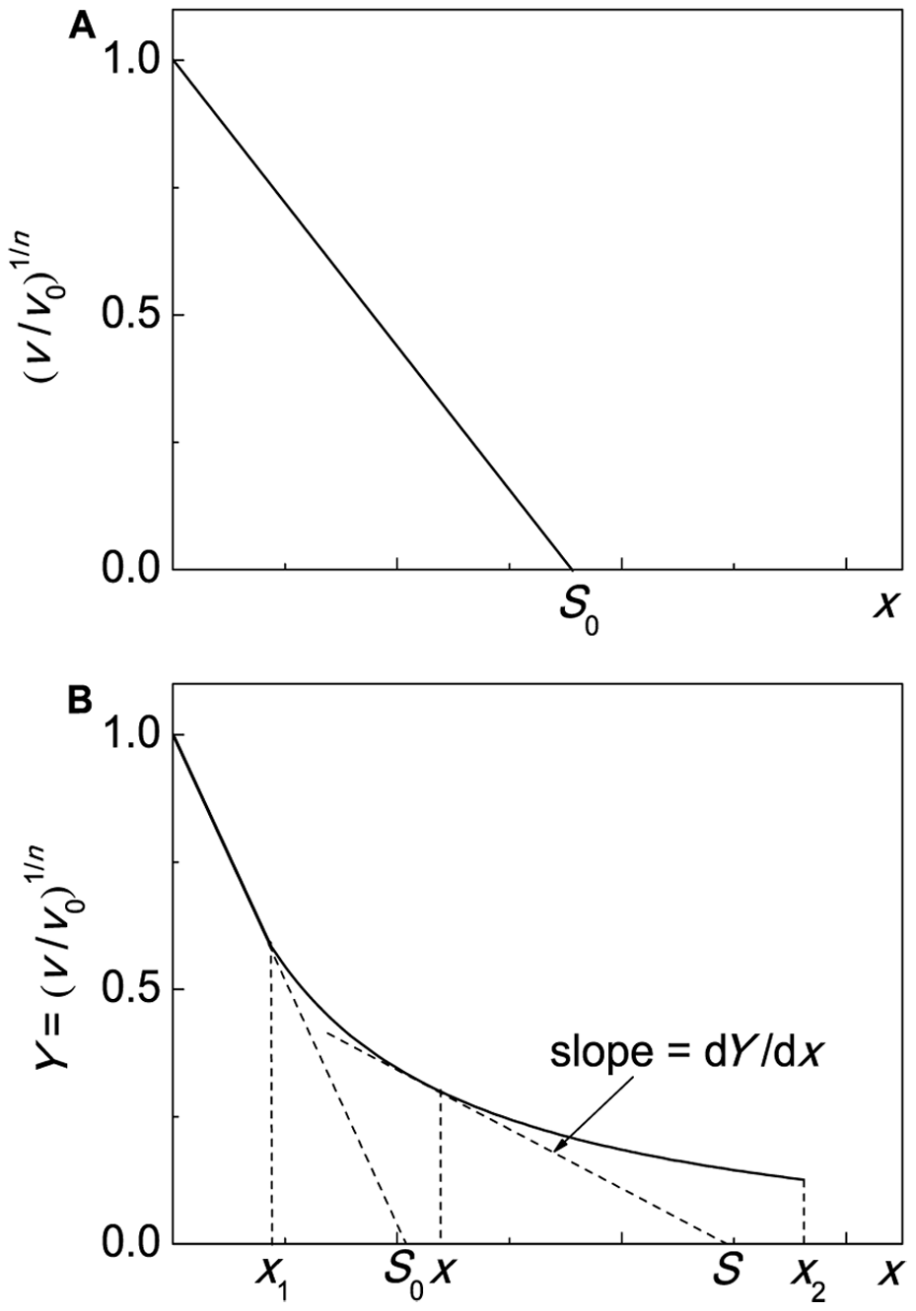
The schematic representation of suppression of protein aggregation by a protein chaperone. The dependences of the relative initial rate of aggregation (*v*/*v*_0_)^1/*n*^ on the ratio of the concentrations of the chaperone and target protein. The following designations are used: *v*_0_ and *v* are the initial rate of aggregation of target protein in the absence and presence of chaperone, respectively; *n* is a power exponent in Eq. (9); *x* is the [chaperone]/[target protein] ratio; *S* is the stoichiometry of the chaperone–target protein complex. (**A**) The formation of the chaperone– target protein complex with constant stoichiometry *S*_0_. (**B**) Case when the stoichiometry of the chaperone–target protein complex is changed with variation of the [chaperone]/[target protein] ratio.

Second, in accordance with Eq. (9) the protein concentration [P]_0_ is proportional to *v* ^1/*n*^. This means that the decrease in the concentration of the target protein (for example, as a result of the complexation with a chaperone) should result in the proportional decrease in the *v* ^1/*n*^ value. Thus, the coordinates {*v* ^1/*n*^; [chaperone]} should be used for analysis of the anti-aggregation activity of the chaperone. The relative initial rate of aggregation *v*/*v*_0_ is determined by the ratio of the concentrations of the chaperone and target protein, namely [chaperone]/[target protein]. Ideally, the dependence of (*v*/*v*_0_)^1/*n*^ on the [chaperone]/[target protein] ratio is a straight line (Fig. 1A). The length on the abscissa axis cut off by the straight line (*S*_0_) gives the stoichiometry of the chaperone–target protein complex. The *S*_0_ value is calculated according to the following equation:
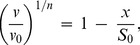
(12)where *x* is the [chaperone]/[target protein] ratio. The reciprocal value of the stoichiometry of the chaperone–target protein complex is the adsorption capacity of the chaperone with respect to the target protein: AC_0_ = 1/*S*_0_. When working with the same test-system, we can use the initial capacity of the chaperone AC_0_ for the comparative analysis of the effectiveness of the anti-aggregation activity of various chaperones (for example, the protective ability of wild-type small heat shock proteins and their mutant forms or the protective ability of the intact chaperone and its chemically modified form).

Consider the dependence of the initial rate of aggregation of UV-irradiated Ph*b* on the αB-crystallin concentration obtained in [22] (37°C; pH 6.8). The *v* value was calculated using Eq. (11). It is significant that the target protein is Ph*b* completely denatured by UV-radiation. The initial part of the dependence of the *v* value on the αB-crystallin concentration gives the following value of AC_0_: AC_0_ = 0.65±0.06 moles of Ph*b* subunit per one αB-crystallin subunit. Interestingly, the deviation from linearity takes place at rather high concentrations of αB-crystallin. The complicated shape of the *v* versus [αB-crystallin] plot is probably due to the dynamic structure of α-crystallin and the initial part of this dependence corresponds to the complexes of the dissociated forms of αB-crystallin with the target protein. The second linear part corresponds to the formation of the αB-crystallin–target protein complexes where the adsorption capacity of αB-crystallin in respect to the target protein becomes decreased.

When the dependence of the initial rate of aggregation on the [chaperone]/[target protein] ratio reveals a deviation from linearity, the following approach may be used for estimation of the stoichiometry of the chaperone–target protein complex. Consider, for example, the case when the initial part of the dependence of the initial rate of aggregation on *x* = [chaperone]/[target protein] gives way to a flatter curve at *x*>*x*_1_, and this flatter part is described by the hyperbolic dependence in the interval of *x* values from *x*_1_ to *x*_2_ (see Fig. 1B):
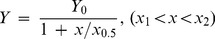
(13)where *Y* signifies (*v*/*v*_0_)^1/*n*^, *Y*_0_ is the *Y* value at *x* = 0, and *x*_0.5_ is the *x* value at which *Y* = *Y*_0_/2. Let us choose some point between *x*_1_ and *x*_2_. It is seen from Fig. 1B that the slope of a tangent to the theoretical curve at the point with coordinates {*x*; *Y*} is connected with the stoichiometry of the chaperone–target protein complex by the following equation:



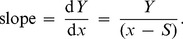
(14)Hence it follows that:
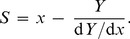
(15)


The derivative d*Y*/d*x* is calculated from Eq. (15):
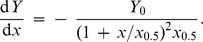
(16)


Substitution of d*Y*/d*x* in Eq. (16) produces the expression, which allows us to calculate the stoichiometry of the chaperone–target protein complex formed at a definite value of *x* in the interval *x*_1_<*x*<*x*_2_:
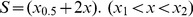
(17)


The adsorption capacity (AC) of the chaperone with respect to the target protein is calculated as a reciprocal value of *S*:
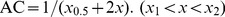
(18)


Thus, in the interval of the *x* values from *x*_1_ to *x*_2_ the value of AC decreases from 1/(*x*_0.5_+2*x*_1_) to 1/(*x*_0.5_+2*x*_2_). As for the initial part of the dependence of (*v*/*v*_0_)^1/*n*^ on the [chaperone]/[target protein ] ratio (the region where *x*<*x*_1_), the adsorption capacity of the chaperone is constant and equal to AC_0_.

### Characterization of Anti-Aggregation Activity of Chemical Chaperones

Protective effect of chemical chaperones is revealed as a diminishing of the initial rate of aggregation (*v*) in the presence chemical chaperone. In the simplest case the dependence of *v* on the concentration of a chemical chaperone (L) is hyperbolic:
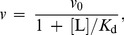
(19)where *v*_0_ is the initial rate of aggregation in the absence of a chaperone and *K*_d_ is the dissociation constant. This equation was applied, for example, by Wilcken et al. [95] for analysis of suppression of p53 oncogenic mutant aggregation by drugs (37°C; pH 7.2). The initial rate of aggregation was calculated using Eq. (3).

When studying the suppression of aggregation of UV-irradiated protein GAPDH by chemical chaperone 2-hydroxylpropyl-β-cyclodextrin [82], we showed that the dependence of the initial rate of aggregation *v* expressed by parameter *k*_agg_ on the concentration of chemical chaperone followed the Hill equation (see [96]):
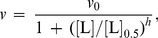
(20)where [L]_0.5_ is the concentration of semi-saturation, i.e., the concentration of the chaperone at which *v*/*v*_0_ = 0.5, and *h* is the Hill coefficient. The value of *h* was found to be 1.8. The values of *h* exceeding unity are indicative of the existence of positive cooperative interactions between chaperone-binding sites in the target protein molecule [96]. Parameter [L]_0.5_ may be considered as a measure of the affinity of the chaperone to the target protein. The lower the [L]_0.5_ value, the higher is affinity of the chaperone to the target protein. It is significant that the shape of the dependence of the initial rate of aggregation on the chaperone concentration should remain unchangeable at variation of the target protein concentration.

### Combined Action of Chaperones

The protective activity of protein chaperones can be modulated by the low-molecular-weight chemical chaperones. For example, it was demonstrated that Arg enhanced the chaperone-like activity of α–crystallin [39], [40], [97]. Since each of the chaperones (protein chaperone or chemical chaperone) affects protein aggregation, strict quantitative methods should be used to characterize the combined action of chaperones. Parameter *j* proposed by us for analysis of combined action of inhibitors [98] may be useful for estimating the mutual inhibitory effects of chaperones:
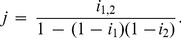
(21)


In this equation *i* is a degree of inhibition: *i*_1_ = 1 − *v*_1_/*v*_0_ for inhibitor 1, *i*_2_ = 1 − *v*_2_/*v*_0_ for inhibitor 2 and *i*_1,2_ = 1 − *v*_1,2_/*v*_0_ for the inhibitor 1+inhibitor 2 mixture (*v*_0_ is the initial rate of aggregation in the absence of inhibitors, *v*_1_, *v*_2_ and *v*_1,2_ are the values of the initial rate of aggregation in the presence of inhibitor 1, inhibitor 2 and inhibitor 1+inhibitor 2 mixture, respectively). When the action of one inhibitor is not dependent on the presence of the other, parameter *j* is equal to unity. The case *j*>1 corresponds to synergism and the case *j*<1 corresponds to antagonism in the combined action of two inhibitors.

As mentioned above, the different parameters are used for characterization of the anti-aggregation activity of protein and chemical chaperones, namely the initial adsorption capacity AC_0_ and the semi-saturation concentration [L]_0.5_, respectively. We can propose the following strategy for the estimation of the effects of the combined action of chaperones. Parameter *j* may be used for this purpose, if we study the mutual effects of chaperones of definite group, i. e., the effects of protein chaperones or the effects of chemical chaperones. In the case of protein chaperone+chemical chaperone mixtures using of parameter *j* becomes unreasonable. To characterize the mutual action of protein and chemical chaperones, we should study the effect of a chemical chaperone on the AC_0_ value for a protein chaperone or the effect of a protein chaperone on the [L]_0.5_ value for a chemical chaperone. A decrease in the AC_0_ value in the presence of a chemical chaperone or a decrease in the [L]_0.5_ value in the presence of a protein chaperone implies synergism in the combined action of protein and chemical chaperones. On the contrary, an increase in the AC_0_ value in the presence of a chemical chaperone or an increase in the [L]_0.5_ value in the presence of a protein chaperone implies antagonism in the combined action of protein and chemical chaperones.

## Results

### Chromatographic and Electrophoretic Analysis of Cross-Linked α-Crystallin Preparation

**Figure 2:**
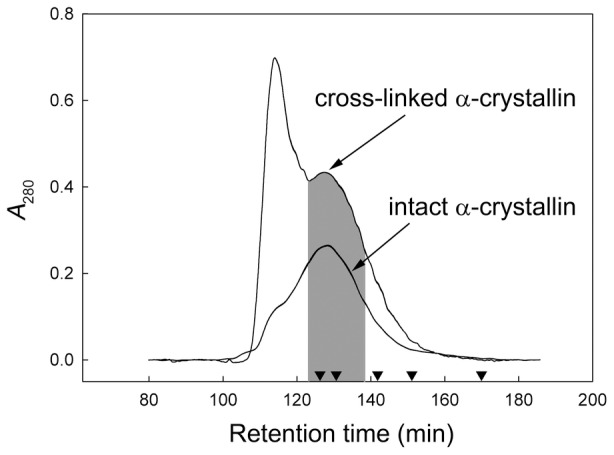
SEC elution profiles of cross-linked and intact α-crystallin on a TSK-gel HW-55f column. The fraction of cross-linked protein marked with gray color was isolated for further testing the chaperone-like activity. Triangles point out retention time of the protein standards: thyroglobulin (660 kDa), catalase (440 kDa), aldolase (158 kDa), BSA (67 kDa), α-crystallin (20 kDa).

Fig. 2 shows the elution profiles obtained for intact and cross-linked α-crystallin by SEC. As can be seen, intact α-crystallin is eluted at 127 min. The cross-linked protein consisting of two fractions is eluted at 107 and 128 min. The peak at 107 min is a high-molecular-weight product of inter-oligomeric cross-linking, whereas the peak at 128 min is a result of intra-molecular cross-linking. The fraction of cross-linked protein marked with gray color (Fig. 2) was collected and examined by SDS electrophoresis in 12.5% PAGE.

**Figure 3:**
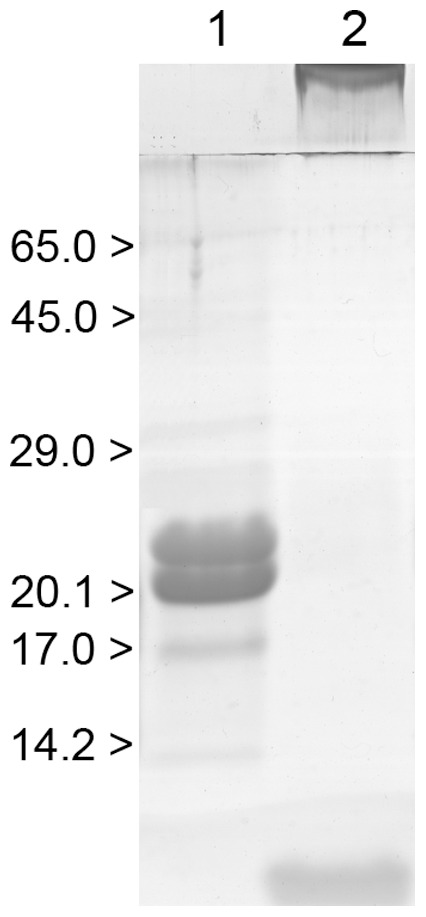
SDS-PAGE of intact α-crystallin (10 µg; line 1) and cross-linked α-crystallin (20 µg; line 2). Relative molecular masses (in kDa) of standard proteins are indicated to the left of lane 1.

Fig. 3 demonstrates the SDS-PAGE patterns for the native and cross-linked α-crystallin. Even when the gel was overloaded (20± µg protein) only traces of low-molecular-weight species can be observed in the cross-linked sample. Most of the protein (99%) was present as high-molecular-weight species, which have not entered the 5% stacking gel.

**Figure 4:**
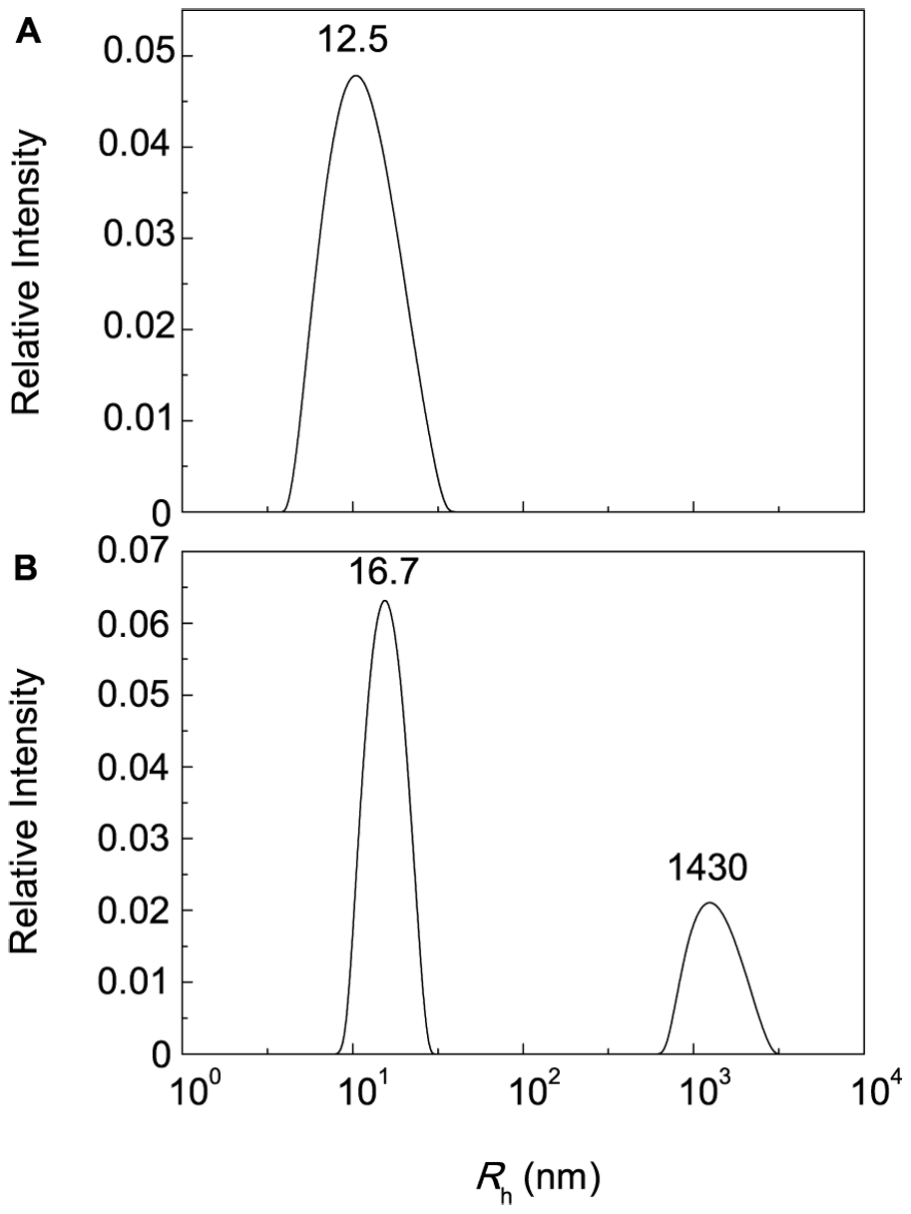
Distribution of the particles by size for intact and cross-linked α-crystallin. The samples of intact (A) α-crystallin (0.1 mg/ml) and (B) cross-linked α-crystallin (0.1 mg/ml) were incubated at 25°C (0.1 M phosphate buffer, pH 7.0).

The preparations of intact and cross-linked α-crystallin were additionally characterized by DLS. The average hydrodynamic radius of intact α-crystallin particles was found to be 12.5 nm (Fig. 4A). The major peak of the particle size distribution for cross-linked α-crystallin has the similar *R*_h_ value (*R*_h_ = 16.7 nm; Fig. 4B). Apart from this peak, there are larger particles with *R*_h_ = 1430 nm.

### Kinetics of DTT-Induced Aggregation of BSA

**Figure 5:**
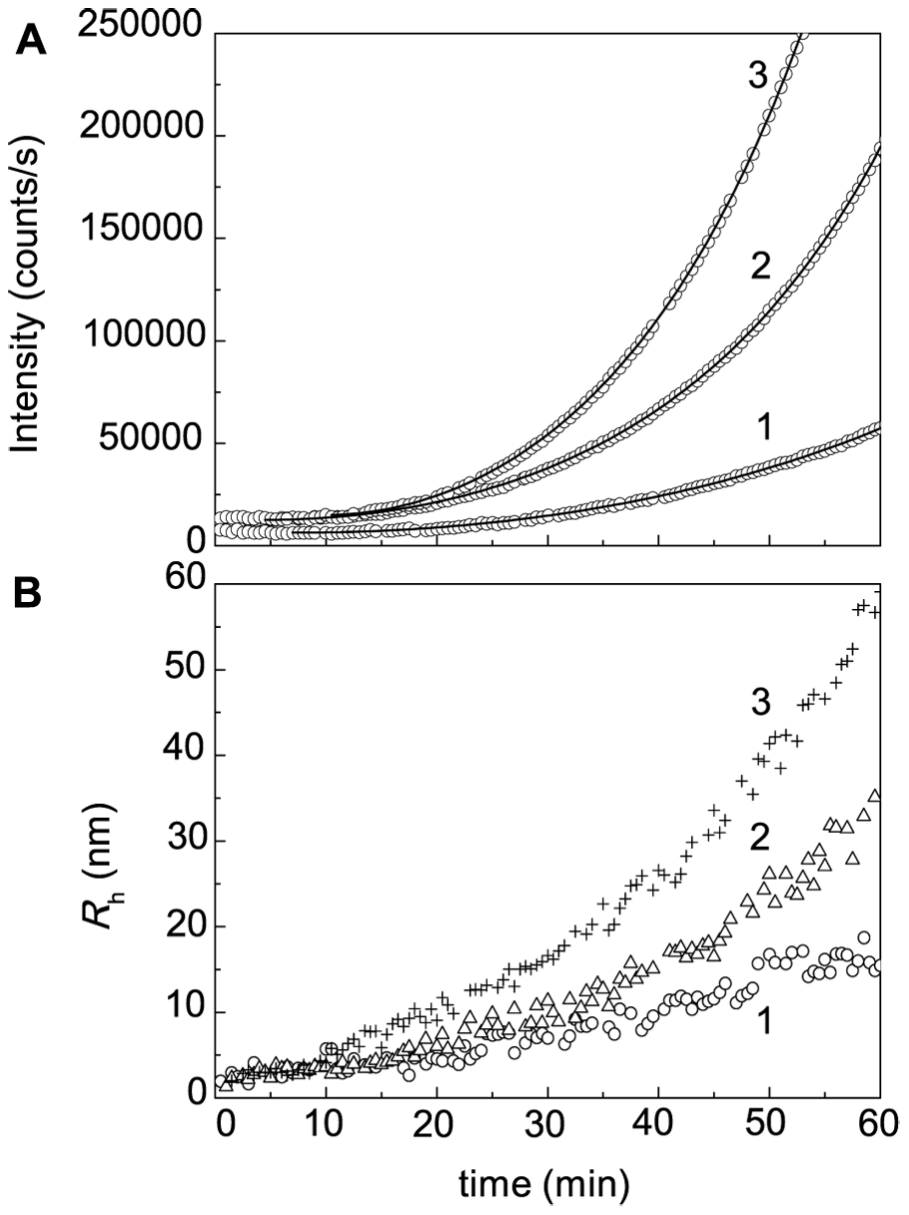
Kinetics of DTT-induced aggregation of BSA. (A) The dependences of the light scattering intensity (*I*) on time obtained at the following concentrations of BSA: (1) 0.5, (2) 1 and (3) 1.5 mg/ml (0.1 M phosphate buffer, pH 7.0; 45°C). The concentration of DTT was 2 mM. Points are the experimental data. Solid curves were calculated from Eq. (3). (B) The dependences of the hydrodynamic radius (*R*_h_) of particles on time registered for heated BSA solutions. BSA concentrations were the following: (1) 0.5, (2) 1.5 and (3) 3 mg/ml.

DLS allows measuring the increment of the light scattering intensity during protein aggregation and sizing the protein aggregates. Fig. 5A shows the dependences of the light scattering intensity on time for DTT-induced aggregation of BSA registered at various concentrations of the protein (45°C; 0.1 M Na-phosphate buffer, pH 7.0; [DTT] = 2 mM).

Fig. 5B shows the dependences of the hydrodynamic radius (*R*_h_) of the protein aggregates on time obtained at various concentrations of BSA. Sizing the protein aggregates by DLS shows that the distribution of the particles by size in the course of DTT-induced aggregation of BSA remains unimodal, and the average value of *R*_h_ increases monotonously with increasing the time of incubation. The value of *R*_h_ for the original preparation of BSA was equal to 4.2±0.1 nm.

The polydispersity index (PI) for BSA particles at 25°C calculated in accordance of the ISO standard [99] was found to be 0.49±0.01. Such a relatively high value of PI is due to the fact that the original preparation of BSA is represented by monomeric and dimeric forms (see [100], [101] and our experimental data given below). The measurements of the PI value for aggregates formed upon heating of BSA (1 mg/ml) in the presence of 2 mM DTT at 45°C were taken over 2 h. It was shown that PI value remained practically constant: PI = 0.51±0.01.

**Figure 6:**
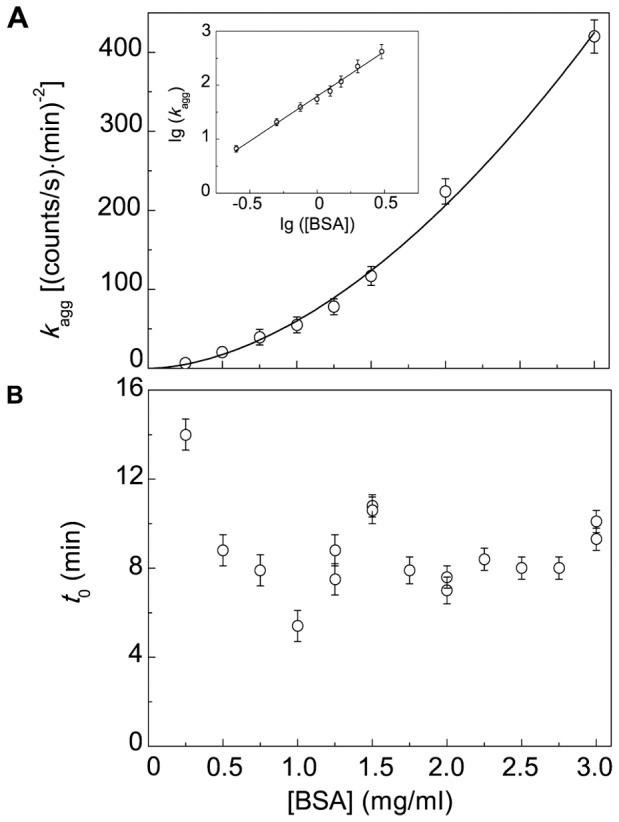
Kinetic parameters for DDT-induced aggregation of BSA (45°C; 2 mM DTT). (A) The dependence of parameter *k*_agg_ on BSA concentration. The solid curve was calculated from Eq. (9) at *n* = 1.6. Inset shows the dependence of *K*_agg_ on BSA concentration in the logarithmic coordinates. (B) The dependence of duration of the lag period (*t*_0_) on BSA concentration.

The initial parts of the dependences of the light scattering intensity on time obtained at various concentrations of BSA were analyzed using Eq. (3). The calculated values of parameters *k*_agg_ and *t*_0_ are represented in Fig. 6 as a function of BSA concentration. As can be seen in Fig. 6A, the dependence of *k*_agg_ on BSA concentration is non-linear. This dependence was treated using Eq. (9). To determine the order of aggregation with respect to the protein (*n*), the plot of lg(*K*_agg_) versus lg([BSA]) was constructed (inset in Fig. 6A). The slope of straight line in these coordinates gives the *n* value: *n* = 1.60±0.05.

When measuring the duration of the lag period, we observed the decrease in the *t*_0_ value from 14±1 to 5.4±0.2 min, as BSA concentration increased from 0.25 to 1.0 mg/ml (Fig. 6B). However, the *t*_0_ value remained practically constant in the interval of BSA concentrations from 1.25 to 3.0 mg/ml (the average value of *t*_0_ was found to be 8.0±0.5 min).

Variation of DTT concentration shows that the initial rate of BSA aggregation is dependent on the concentration of disulfide reducing agent. The increase in DTT concentration from 1.3 to 3.3 mM results in the increase in the *k*_agg_ value from 29.1±0.5 to 135±2 [(counts/s) (min)^−2^] at [BSA] = 1.0 mg/ml.

### Effects of Intact and Cross-Linked α-Crystallin on DTT-Induced Aggregation of BSA

**Figure 7:**
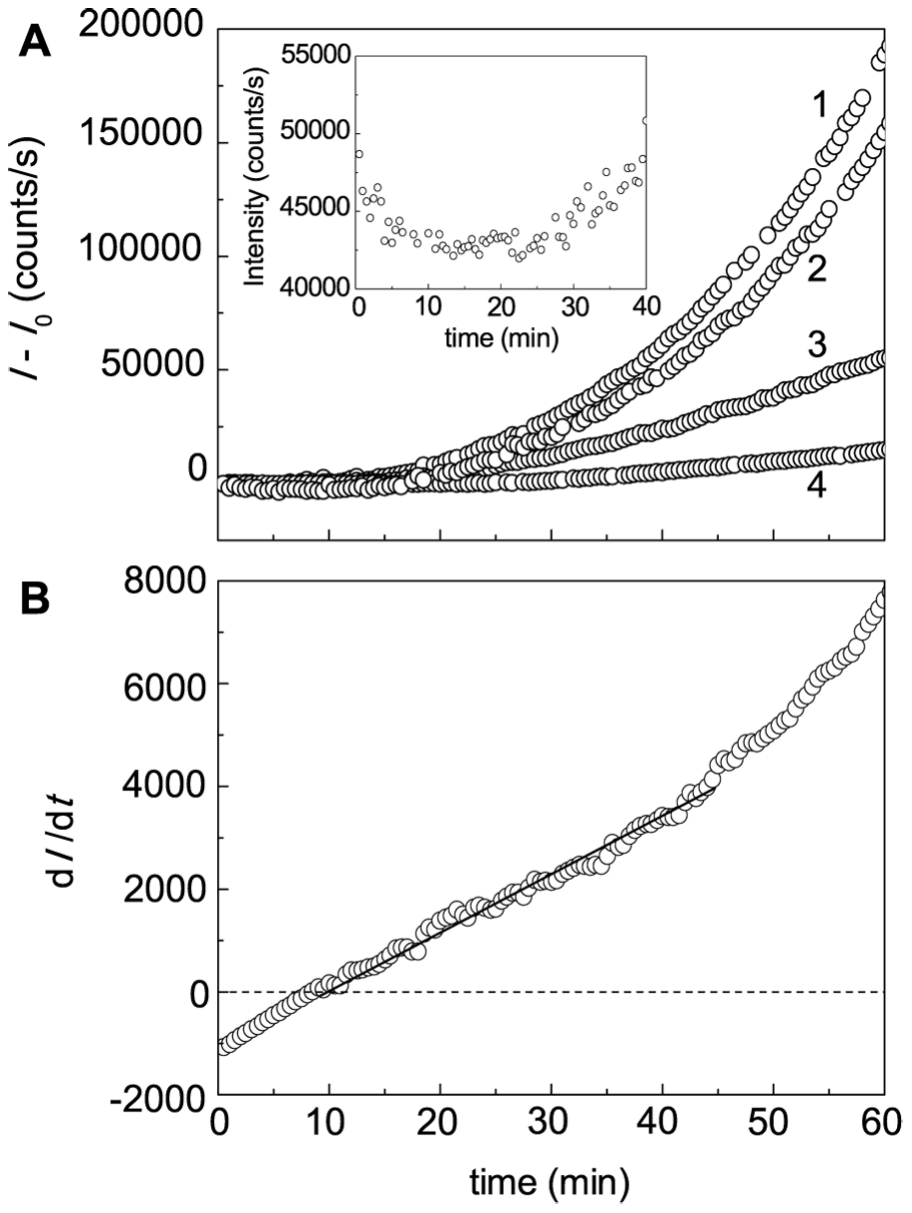
Effect of α-crystallin on DTT-induced aggregation of BSA. (A) The dependences of the light scattering intensity on time obtained at the following concentrations of α-crystallin: (1) 0, (2) 0.01, (3) 0.1 and (4) 1 mg/ml ([BSA] = 1.0 mg/ml; 2 mM DTT; 0.1 M phosphate buffer, pH 7.0; 45°C). *I* and *I*_0_ are the current and initial values of the light scattering intensity, respectively. The inset shows the dependence of *I* on time obtained at the concentration of α-crystallin 0.5 mg/ml. (B) The dependence of d*I*/d*t* on time at α-crystallin concentration of 0.05 mg/ml. Points are the experimental data. The solid curve was calculated from Eq. (7).

As can be seen from Fig. 7A, α-crystallin suppresses DTT-induced aggregation of BSA. In this Figure the dependences (*I*-*I*_0_) on time are represented (*I* and *I*_0_ are the current and initial values of the light scattering intensity, respectively). When the reaction mixture contains α-crystallin, the initial decrease in the light scattering intensity on the kinetic curves of aggregation is observed. For example, inset in Fig. 7A shows the initial part of the kinetic curve obtained at α-crystallin concentration of 0.5 mg/ml. This circumstance poses difficulties for using of Eq. (3) for calculation of the initial rate of aggregation, because determination of the *I*_0_ value becomes impossible. Therefore, to determine parameter *k*_agg_ characterizing the initial rate of aggregation, we apply the differential form of Eq. (3), namely, Eq. (7). In accordance with Eq. (7), the slope of the straight line for the initial positive values of d*I*/d*t* gives the 2 *k*_agg_ value (Fig. 7B; [α-crystallin] = 0.05 mg/ml).

**Figure 8:**
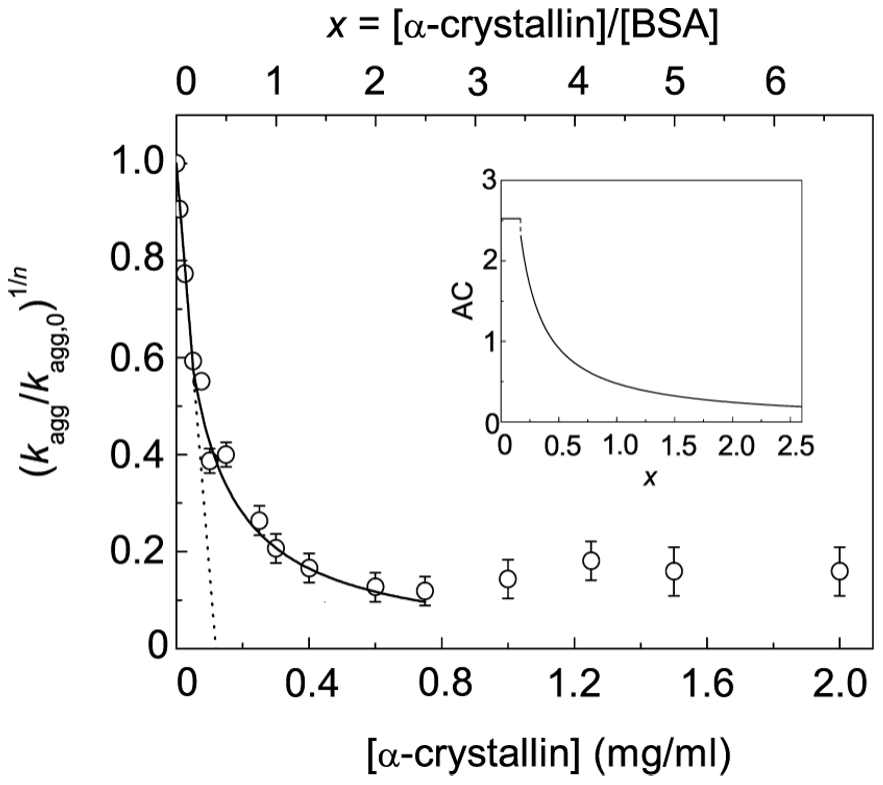
Initial rate of DDT-induced aggregation of BSA as a function of α-crystallin concentration (45°C; 2 mM DTT). The dependence of (*K*_agg_/*K*_agg,0_)^1/*n*^ on α-crystallin concentration (lower abscissa axis) or the ratio of the molar concentrations of α-crystallin and BSA (upper abscissa axis *x* = [α-crystallin]/[BSA]; *n* = 1.6). Points are the experimental data. The solid line in the interval of *x* values from 0 to *x*_1_ = 0.17 was calculated from Eq. (12) at *S*_0_ = 0.40 subunits of α-crystallin per one BSA molecule. The solid line in the interval of *x* values from *x*_1_ = 0.17 to *x*_2_ = 2.6 was calculated from Eq. (13) at *Y*_0_ = 0.94 and *x*_0.5_ = 0.093. The inset shows the dependence of the adsorption capacity (AC) of α-crystallin with respect to the target protein on *x*.

The *k*_agg_ values were calculated at various concentrations of α-crystallin, and the plot of (*k*_agg_/*k*_agg,0_)^1/*n*^ versus α-crystallin concentration was constructed (Fig. 8). The additional abscissa axis is shown in this Figure: *x* = [α-crystallin]/[BSA], where [α-crystallin] is the molar concentration of α-crystallin calculated on subunit and [BSA] is the molar concentration of BSA. In the interval of *x* values from 0 to *x*_1_ = 0.17 the dependence of (*k*_agg_/*k*_agg,0_)^1/*n*^ on *x* is linear. Using Eq. (12) we estimated the stoichiometry of the initial complexes α-crystallin–target protein: *S*_0_ = 0.40±0.01 α-crystallin subunits per one BSA molecule. Knowing the *S*_0_ value, we can calculate the initial adsorption capacity of α-crystallin with respect to the target protein: AC_0_ = 1/*S*_0_ = 2.50±0.06 BSA monomers per one α-crystallin subunit. At *x*>*x*_1_ the dependence of (*k*_agg_/*k*_agg,0_)^1/*n*^ on *x* becomes non-linear and follows the hyperbolic law (Eq. (13) in the interval of the *x* values from *x*_1_ = 0.17 to *x*_2_ = 2.6. Fitting of Eq. (13) to the experimental data gave the following values of parameters: *Y*_0_ = 0.94±0.17 and *x*_0.5_ = 0.093±0.029. In accordance with Eq. (18), the AC value (the adsorption capacity of α-crystallin with respect to the target protein) decreases from 2.33 to 0.19 BSA monomers per one α-crystallin subunit in the interval of *x* values from *x*_1_ = 0.17 to *x*_2_ = 2.6 (inset in Fig. 8). It should be noted that at *x*>*x*_2_ = 2.6 α-crystallin is incapable of completely suppressing DTT-induced BSA aggregation.

**Figure 9:**
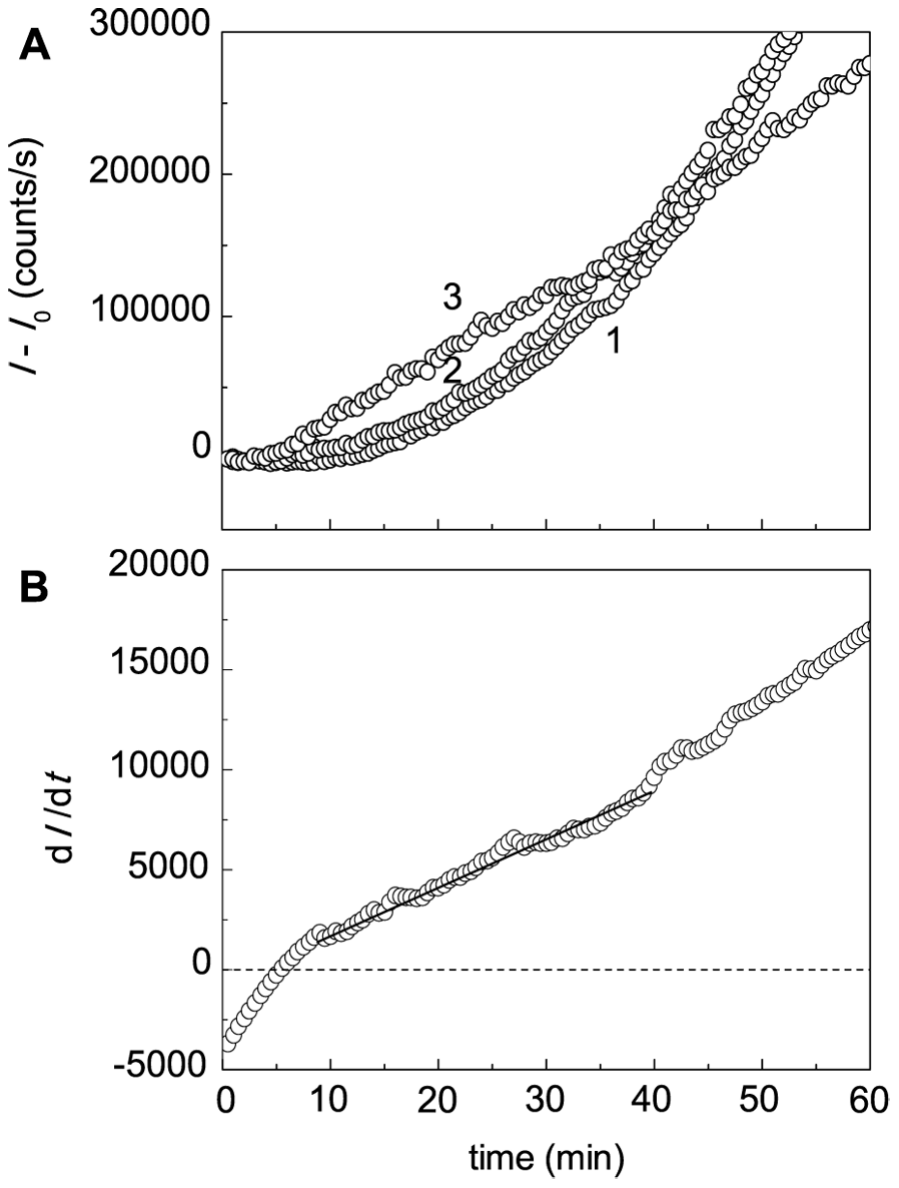
Effect of cross-linked α-crystallin on DTT-induced aggregation of BSA. (A) The dependences of the light scattering intensity on time obtained at the following concentrations of cross-linked α-crystallin: (1) 0, (2) 0.1 and (3) 1.0 mg/ml ([BSA] = 1.0 mg/ml; 2 mM DTT; 0.1 M phosphate buffer, pH 7.0; 45°C). *I* and *I*_0_ are the current and initial values of the light scattering intensity, respectively. (B) The dependence of d*I*/d*t* on time at concentration of cross-linked α-crystallin 0.05 mg/ml. Points are the experimental data. The solid curve was calculated from Eq. (7).

**Figure 10:**
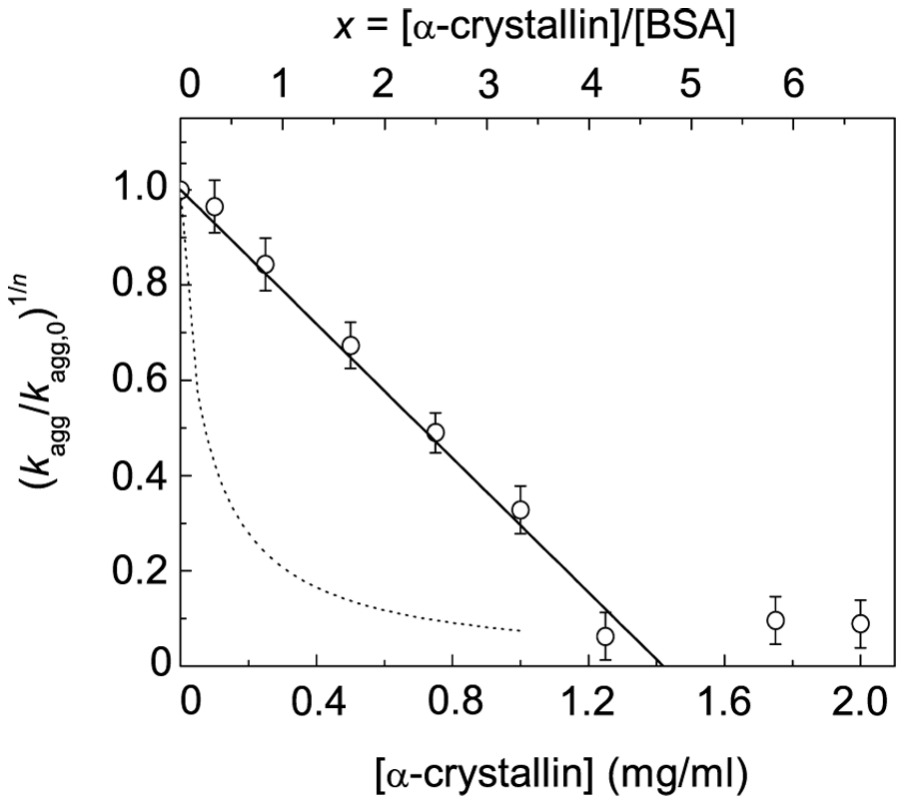
Iinitial rate of DDT-induced aggregation of BSA at 45°C as a function of cross-linked α-crystallin concentration. The solid line was calculated from Eq. (12) at *S*_0_ = 4.7 subunits of α-crystallin per one BSA molecule. The dotted line corresponds to the dependence of (*K*_agg_/*K*_agg,0_)^1/*n*^ on concentration of intact α-crystallin (*n* = 1.6).

When studying the effect of cross-linked α-crystallin on DTT-induced aggregation of BSA (Fig. 9A), we also used Eq. (7) for calculation of parameter *k*_agg_ (Fig. 9B). Fig. 10 shows the dependence of (*k*_agg_/*k*_agg,0_)^1/*n*^ on the concentration of cross-linked -crystallin or *x* = [-crystallin]/[BSA]. As can be seen from this Figure, the noted dependence is linear. Using Eq. (12) allowed us to determine the stoichiometry of the α-crystallin–target protein complex (*S*_0_ = 4.7±0.1 α-crystallin subunits per one BSA molecule) and adsorption capacity of α-crystallin (AC_0_ = 0.212±0.004 BSA monomers per one α-crystallin subunit). It is significant that *S*_0_ and AC_0_ remain constant at variation of the [α-crystallin]/[BSA] ratio.

### Study of DTT-Induced Aggregation of BSA by Asymmetric Flow Field Flow Fractionation (A4F)

**Figure 11:**
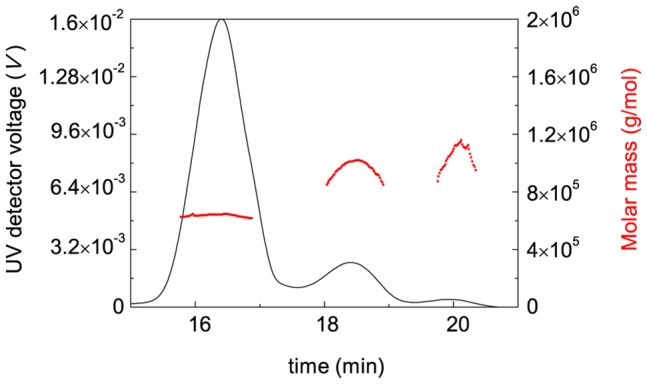
AF4-MALS analysis of BSA (1 mg/ml) at 25°C (0.1 M phosphate buffer, pH 7.0). Molar mass versus elution time plot (points) is overlaid on the UV detector fractogram (solid line). A4F conditions: axial (detector) flow 1 ml/min, focus flow 5 ml/min, cross flow 5 ml/min for 14 min and then linear decay to 0.1 ml/min within 20 min plus 8 min at 0 ml/min.

Fig. 11 shows the elution profile of BSA (1 mg/ml; 25°C) registered as a time-dependence of UV detector signal. As one can see, the sample profile runs broad range of elution times indicating the polydispersity of the size of BSA particles: three distinct peaks appear in this fractogram. According to the values of the molar mass calculated from the MALS data, the detected peaks correspond to the monomeric (*M* = 64.5 kDa), dimeric (*M* = 101.2 kDa) and trimeric forms of BSA. Peak deconvolution gives the following values for the portions of monomer, dimer and trimer: 0.85, 0.14 and 0.01, respectively.

**Figure 12:**
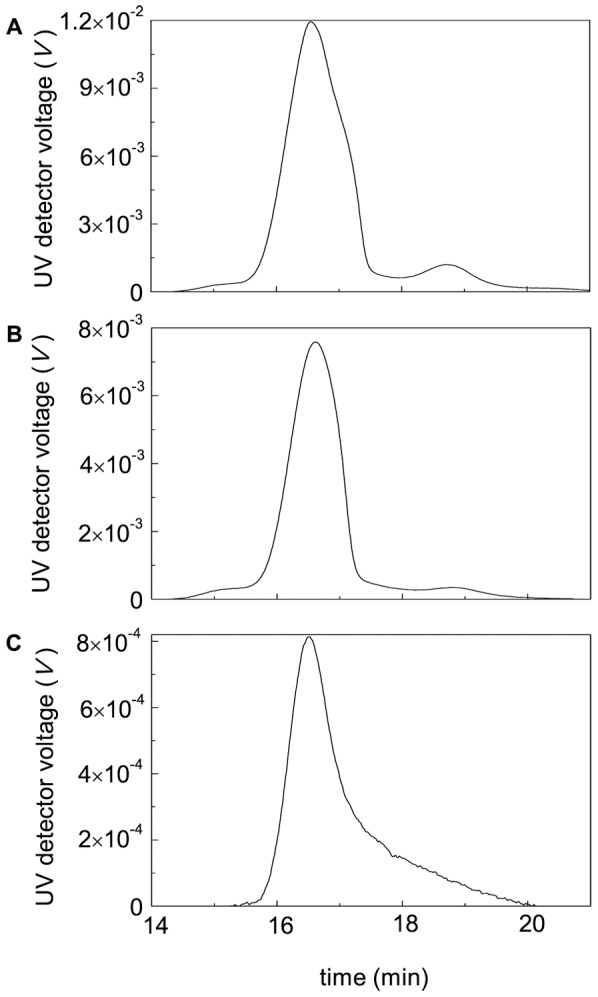
Fractograms of BSA (1 mg/ml) heated at 45°C in the presence of 2 mM DTT. The heating times were the following: 20 (A), 45 (B) and 90 (D) min. AF4 conditions were the same as described in legend to Fig. 11.

**Figure 13:**
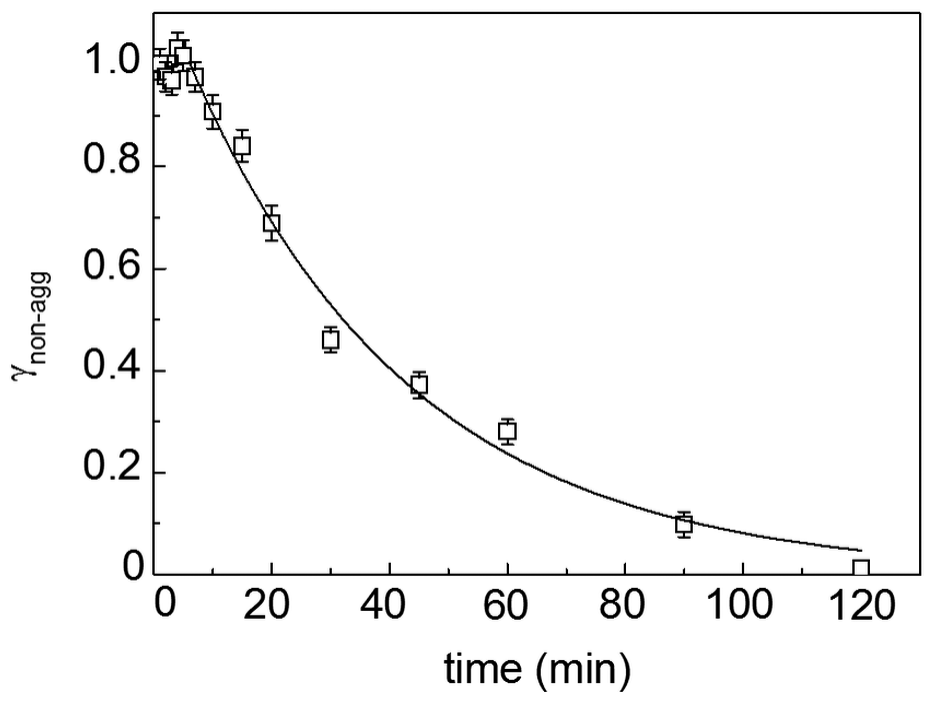
Decrease in the portion of non-aggregated protein (γ_non-agg_) in the course of DTT-induced aggregation of BSA (1 mg/ml) at 45°C. Points are the experimental data. The solid curve was calculated from Eq. (22) at *t*_0_ = 6.2 min and *k*_I_ = 0.027 min^−1^.

Fig. 12 demonstrates the fractograms of BSA heated at 45°C in the presence of 2 mM DTT for different intervals of time (20, 45 and 90 min). Based on the measurements of the area under fractograms, we have constructed the dependence of the portion of the non-aggregated protein (γ_non-agg_) on time (Fig. 13). Analysis of the data shows that the dependence of γ_non-agg_ on time obeys the exponential law of the following type:
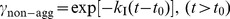
(22)where *k*_I_ is the rate constant of the first order and *t*_0_ is the duration of the lag period. Fitting of the experimental data to this equation gives the following values of parameters: *t*_0_ = 6.2±0.6 min and *k*_1_ = 0.027±0.001 min^−1^. Thus, at *t*>*t*_0_ the decrease of the portion of the non-aggregated protein in time follows the exponential law with *k*_1_ = 0.027 min^−1^. As expected, the *t*_0_ value calculated from Eq. (22) (*t*_0_ = 6.2±0.6 min) is close to the duration of the lag period determined from the kinetic curve of aggregation at [BSA] = 1 mg/ml (*t*_0_ = 5.4±0.2 min).

The exponential decrease in the portion of the non-aggregated protein in time seemingly indicates that any monomolecular stage (conformational transition or protein unfolding) is the rate-limiting stage of the aggregation process. However, our data show that the rate constant *k*_1_ depends on the initial protein concentration. For example, at [BSA] = 2 mg/ml the *k*_1_ value was found to be 0.036±0.001 min^−1^ (data not presented). This means that a two-fold increase in the protein concentration results in the increase of the *k*_1_ value by the factor of 1.32±0.04. Thus, based on the data on BSA aggregation kinetics, where the order with respect to protein was found to be 1.6, and data on AF4 we may conclude that DTT-induced aggregation of BSA can not be classified as a process with monomolecular rate-limiting stage.

### Study of Interaction of BSA Unfolded in the Presence of DTT with α-Crystallin by Analytical Ultracentrifugation

**Figure 14:**
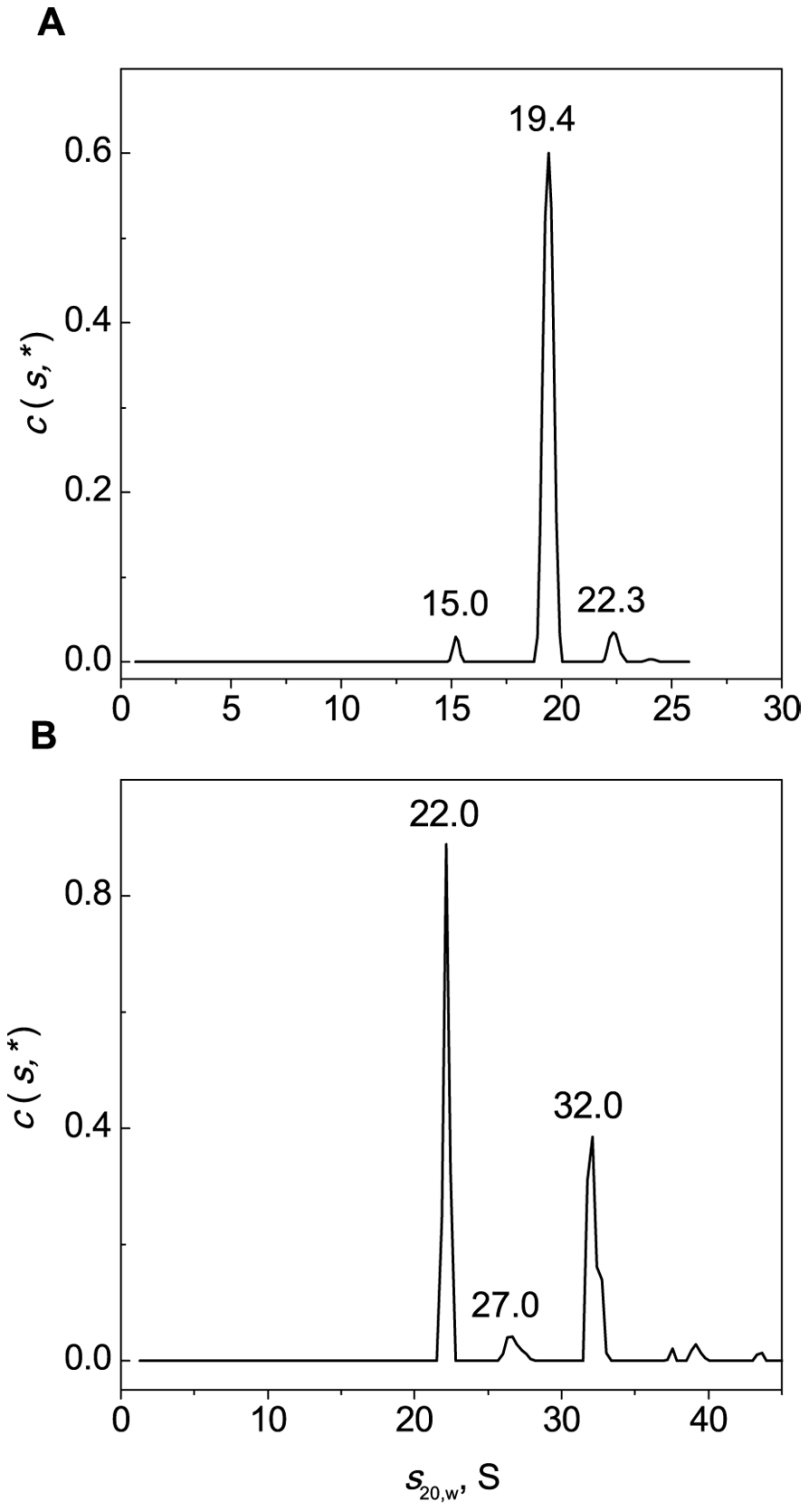
Sedimentation velocity analysis of intact α-crystallin (0.5 mg/ml; A) and cross-linked α-crystallin (0.5 mg/ml; B) heated at 45°C for 1 h. General sedimentation coefficient distributions *c*(*s*,*) obtained at 45°C were transformed to standard *s*_20,w_-distributions. The rotor speed was 34000 rpm.

Additional information on the interaction of BSA unfolded in the presence of DTT with α-crystallin was obtained by analytical ultracentrifugation. Before analyzing the mixtures of BSA and α-crystallin we studied the sedimentation behavior of intact and cross-linked α-crystallin heated at 45°C for 1 h in the presence of 2 mM DTT. The general *c*(*s*,*) distribution for heated α-crystallin (Fig. 14A), besides the major peak with *s*_20,w_ = 19.4 S, revealed two minor peaks (*s*_20,w_ = 15 and 22.3 S). As in the case of intact α-crystallin, cross-linked α-crystallin contained a set of oligomeric forms with the major species with *s*_20,w_ = 22 S (Fig. 14B). It should be noted that small oligomers with *s*_20,w_<21 S were lacking.

**Figure 15:**
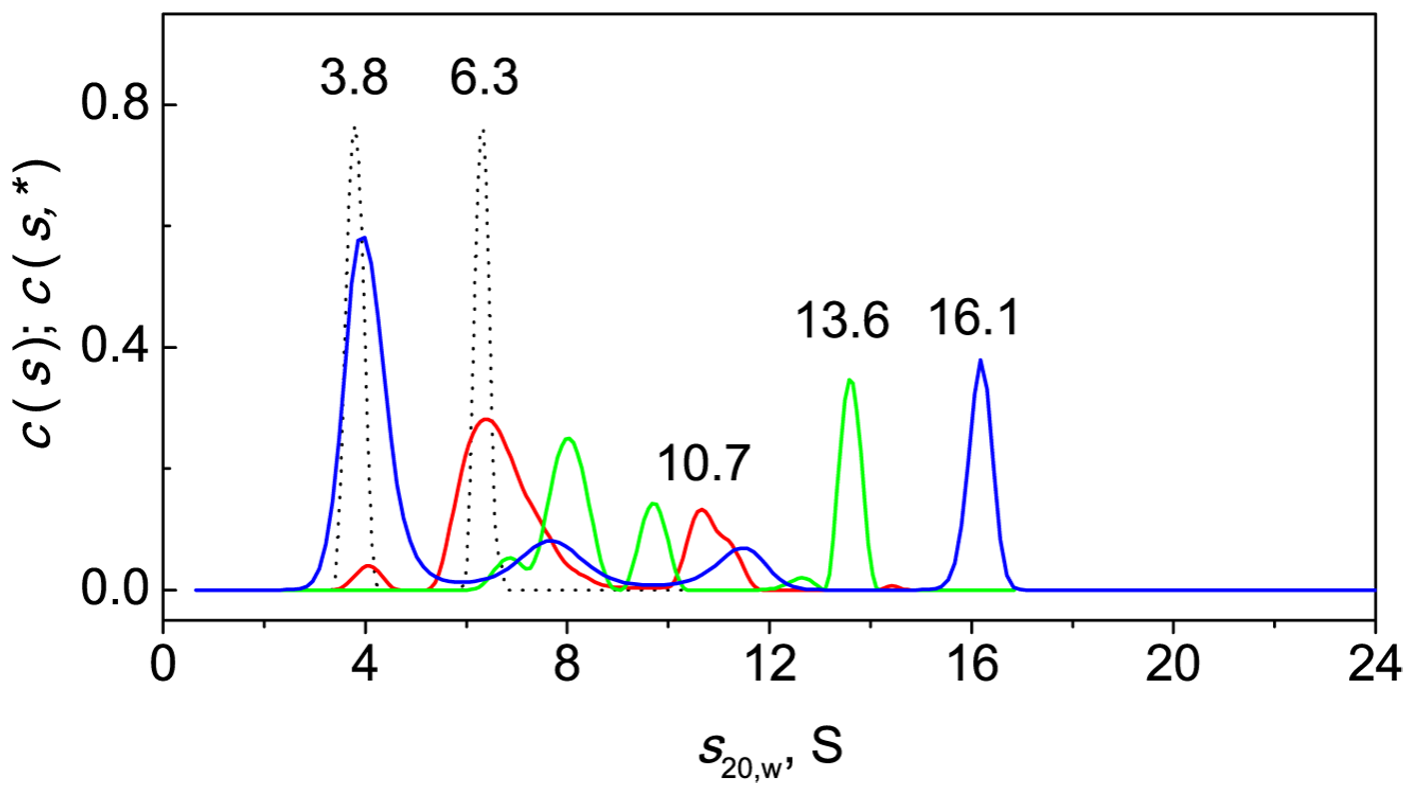
Interaction of BSA with intact α-crystallin upon heating at 45°C. All the samples of BSA (1 mg/ml) and the mixtures of BSA with α-crystallin were heated at 45°C for 1 h in the presence of 2 mM DTT. The *c*(*s*) distributions for BSA (black line) and the mixtures of BSA with α-crystallin at various concentrations (0.05 mg/ml, red line; 0.1 mg/ml, green line; 0.4 mg/ml, blue line) and *c*(*s*,*) for BSA (black dotted line) obtained at 45°C were transformed to standard *s*_20,w_-distributions. The rotor speed was 34000 rpm.

**Figure 16:**
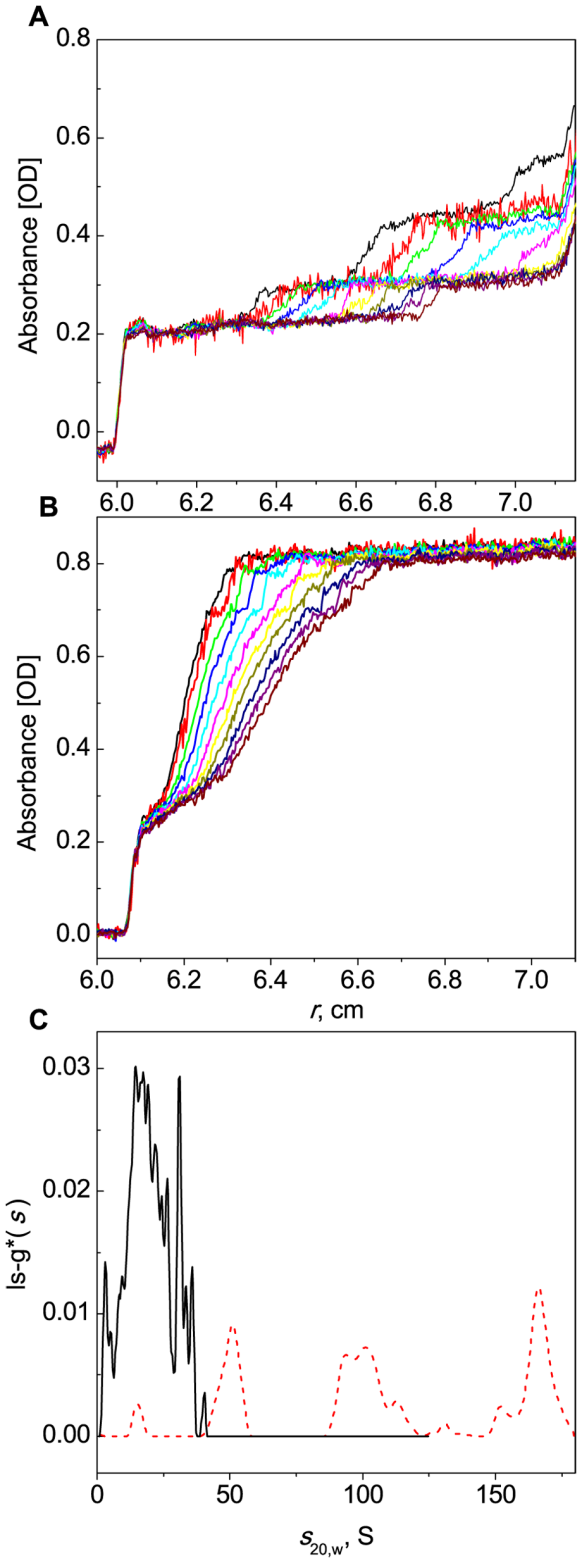
Protection of BSA aggregation by α-crystallin studied by analytical ultracentrifugation. The sedimentation velocity analysis of BSA samples (1 mg/ml) which were held at 45°C for 3.5 h in the absence (A) or in the presence of α-crystallin (0.4 mg/ml; B). Sedimentation profiles were registered at 285 nm with 2.5 min intervals. Selected profiles obtained with 5 min intervals are shown in panels A and B. The rotor speed was 34000 rpm. (C) The differential sedimentation coefficient distributions ls-g*(*s*) for BSA (red dash line) and the mixture of BSA and α-crystallin (black solid line) were calculated from the data presented in panel A and B.

Fig. 15 shows the *c*(*s*) distribution for the mixtures of BSA (1 mg/ml) and α-crystallin at various concentrations (0.05, 0.1 and 0.4 mg/ml). The mixtures were heated at 45°C for 1 h. It is noteworthy that in the case of a mixture of BSA and α-crystallin at the concentration of 0.05 mg/ml (see Fig.15, red line) the *c*(*s*) distribution did not exhibit species for unbound α-crystallin due to its small concentration. A comparison of distributions for BSA (dotted line) and mixture of BSA with α-crystallin (0.05 mg/ml; red line) suggests that the broad peak with average sedimentation coefficient 10.7 S for the mixture corresponds to the complex of chaperone with BSA. A similar comparison of *c*(*s*,*) distribution for BSA and *c*(*s*) distributions for the mixtures of the protein and α-crystallin at higher concentrations indicates that the additional peaks with sedimentation coefficients in the range from 6.8 to 14.5 S may correspond to the BSA–α-crystallin complexes. At the highest concentration of α-crystallin (0.4 mg/ml) the peak with *s*_20,w_ = 16.1 S in *c*(*s*) distribution may correspond to the unbound chaperone and its complex with BSA. It is important to note that the complexes with *s*_20,w_ in the range 6.8–14.5 S were formed by dissociated species of α-crystallin and BSA (compare *c*(*s*) distributions for mixtures with *c*(*s*) distribution data for α-crystallin in Fig. 16A, where species with *s*_20,w_ smaller than 15 S were lacking).

It was interesting to study the anti-aggregation ability of α-crystallin in the case of long-term exposure to 45°C. The protective effect of α-crystallin heated with BSA at 45°C for 3.5 h is demonstrated in Fig. 16. Comparison of the ls-g*(*s*) distribution for BSA with that for the mixture of BSA and α-crystallin revealed that samples with *s*_20,w_ exceeding 50 S were lacking in the ls-g*(*s*) distribution for the mixture (Fig. 16C). Thus, the comparison of the sedimentation profiles of BSA in the absence (A) and in the presence of α-crystallin (B) and ls-g*(*s*) distributions obtained from these data is indicative of the anti-aggregation effect of α-crystallin.

**Figure 17:**
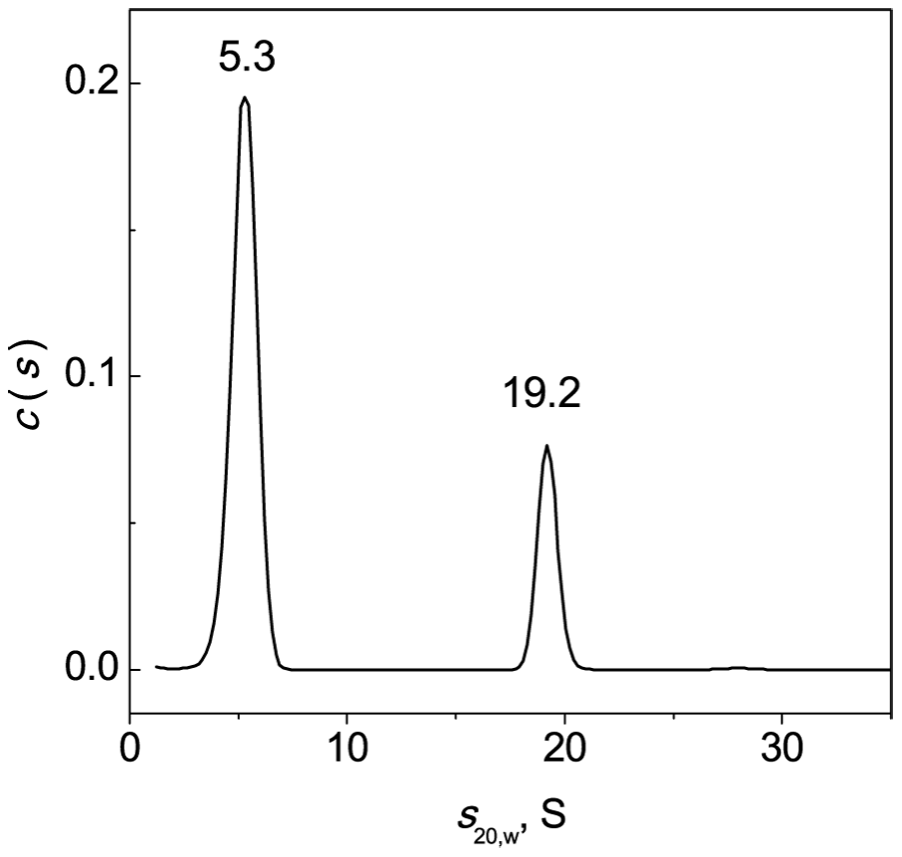
Interaction of BSA with cross-linked α-crystallin upon heating at 45°C. All the samples were heated at 45°C for 1 h in the presence of 2 mM DTT. The *c*(*s*) distribution for the mixture of BSA (1 mg/ml) and α-crystallin (0.05 mg/ml) was transformed to standard *s*_20,w_-distributions. The rotor speed was 34000 rpm.

We also studied the interaction of BSA (1 mg/ml) with cross-linked α-crystallin (0.05 mg/ml) at 45°C. The *c*(*s*) distribution revealed two main peaks with *s*_20,w_ equal to 5.3 and 19.2 S (Fig. 17). We supposed that the major peak with 5.3 S corresponded to BSA. It will be noted that the *c*(*s*) data do not reveal species corresponding to unbound cross-linked α-crystallin. Cross-linked α-crystallin does not contribute to sedimentation profiles due to its low concentration (0.05 mg/ml). Analysis of the *c*(*s*,*) and *c*(*s*) plots in Figs. 14B, 15 (dotted line) and 17 allowed us to conclude that the peak at 19.2 S in Fig. 17 corresponded to the complex of BSA with cross-linked α-crystallin. Samples with *s*_20,w_ in the range 6.8–14 S were lacking (Fig. 17). Thus, in the case of cross-linked α-crystallin the complexes with dissociated forms of α-crystallin were not formed.

### Effect of Chemical Chaperones on DTT-Induced Aggregation of BSA

**Figure 18:**
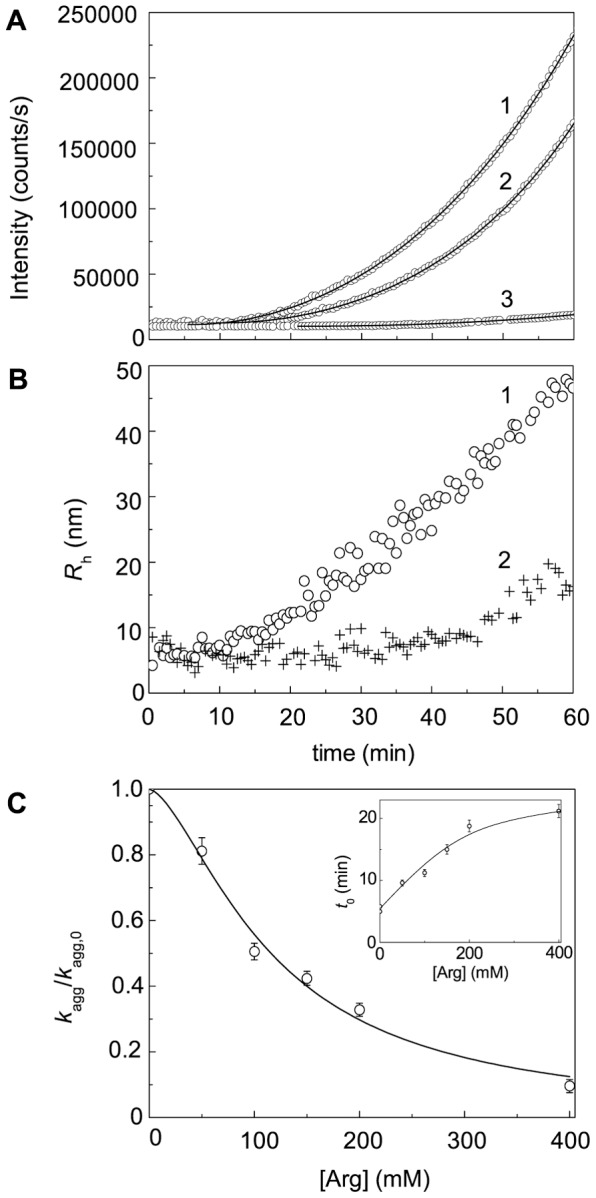
Effect of Arg on DTT-induced aggregation of BSA ([BSA] = 1.0 mg/ml, 2 mM DTT). (A) The dependences of the light scattering intensity on time obtained at the following concentrations of Arg: (1) 0, (2) 50 and (3) 400 mM. Points are the experimental data. The solid curves was calculated from Eq. (5). (B) The dependences of the hydrodynamic radius (*R*_h_) of the protein aggregates on time obtained in the absence of Arg (1) and in the presence of 400 mM Arg (2). (C) The dependence of the *K*_agg_/*K*_agg,0_ ratio on the concentration of Arg. Points are the experimental data. The solid curve was calculated from Eq. (20). Inset shows the dependence of the duration of the lag period (*t*_0_) on the concentration of Arg.

**Figure 19:**
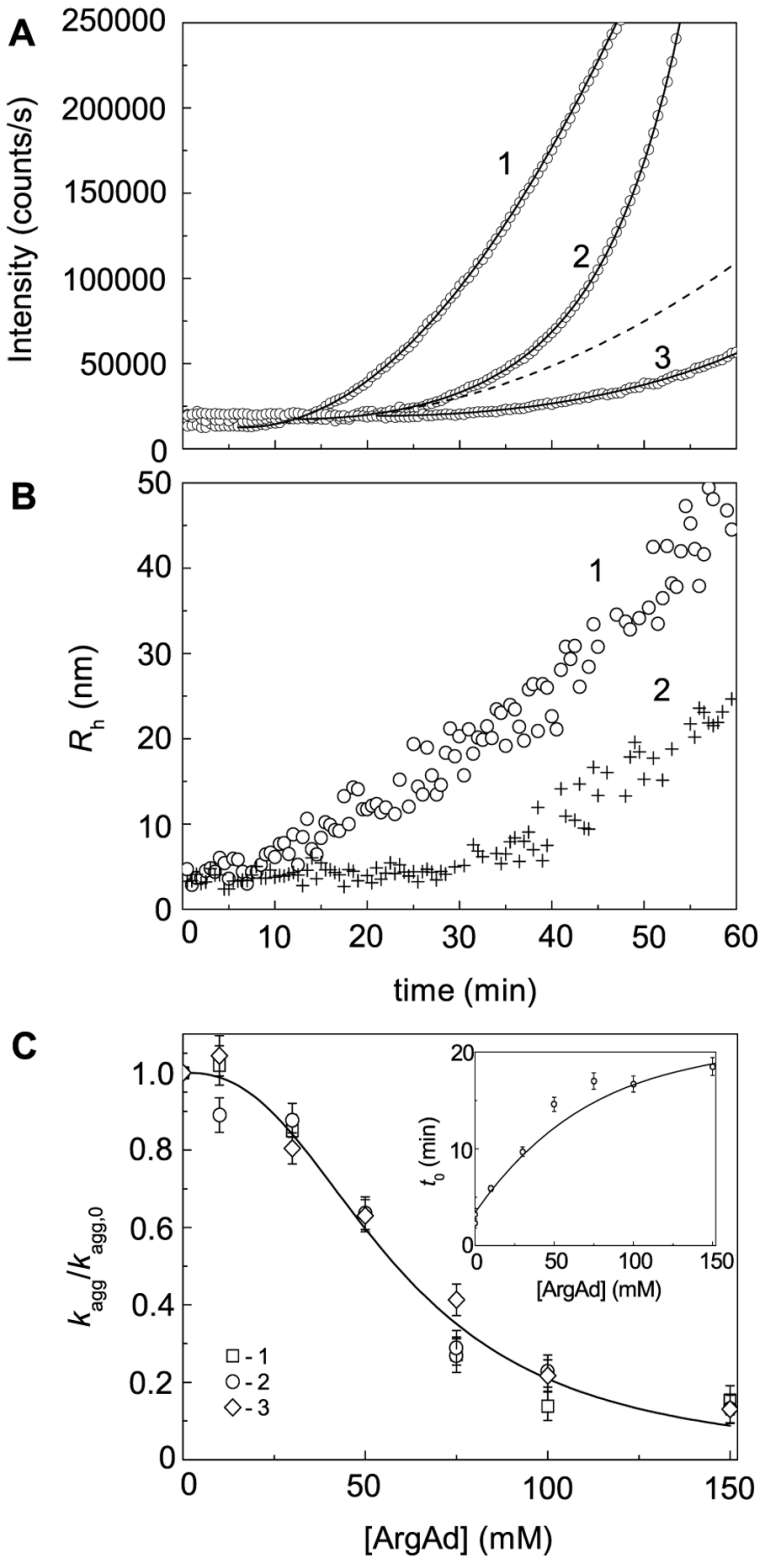
Effect of ArgAd on DTT-induced aggregation of BSA ([BSA] = 1.0 mg/ml; 2 mM DTT). (A) The dependences of the light scattering intensity on time obtained at the following concentrations of ArgAd: (1) 0, (2) 75 and (3) 150 mM. Points are the experimental data. The solid curves were calculated from Eq. (5). At [ArgAd] = 75 mM the fitting procedure gave the following values of parameters: *k*_agg_ = 40.1 (counts/s) min^−2^, *t*_0_ = 12.1 min and *K* = 1.18·10^−3^ min^−2^. The dotted line was calculated from Eq. (3) at *k*_agg_ = 40.1 (counts/s) min^−2^ and *t*_0_ = 12.1 min. (B) The dependences of the hydrodynamic radius (*R*_h_) of the protein aggregates on time obtained in the absence of ArgAd (1) and in the presence of 150 mM ArgAd (2). (C) The dependence of the *K*_agg_/*K*_agg,0_ ratio on the concentration of ArgAd. Points are the experimental data corresponding to the following concentrations of BSA: (1) 0.5, (2) 1 and (3) 2 mg/ml. The solid curve was calculated from Eq. (20). Inset shows the dependence of the duration of the lag period (*t*_0_) on the concentration of ArgAd.

**Figure 20:**
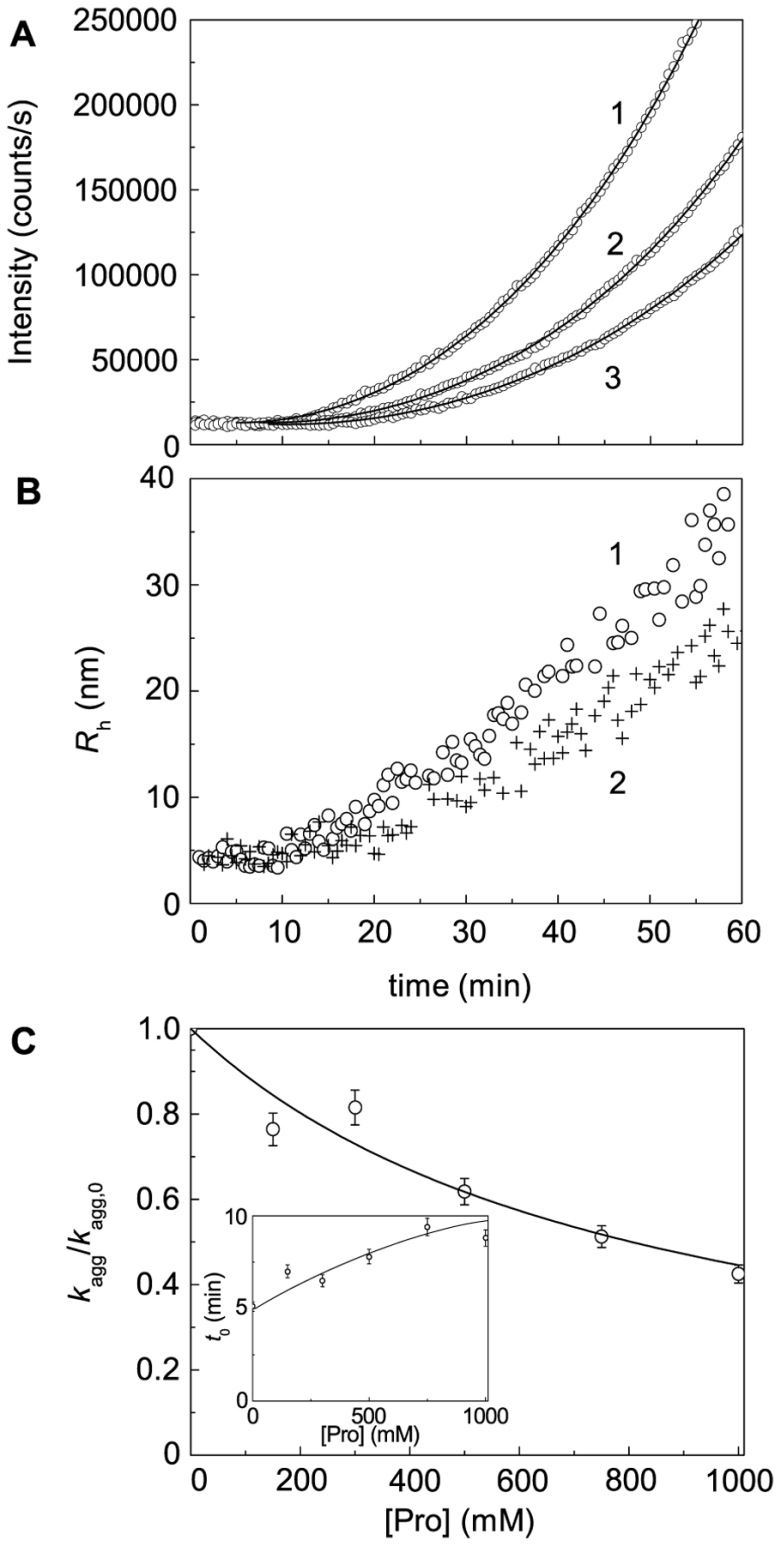
Effect of Pro on DTT-induced aggregation of BSA ([BSA] = 1.0 mg/ml, 2 mM DTT). (A) The dependences of the light scattering intensity on time obtained at the following concentrations of Pro: (1) 0, (2) 500 and (3) 1000 mM. Points are the experimental data. The solid curves were calculated from Eq. (5). (B) The dependences of the hydrodynamic radius (*R*_h_) of the protein aggregates on time obtained in the absence of Pro (1) and in the presence of 1000 mM Pro (2). (C) The dependence of the *K*_agg_/*K*_agg,0_ ratio on the concentration of Pro. Points are the experimental data. The solid curve was calculated from Eq. (20). Inset shows the dependence of the duration of the lag period (*t*_0_) on the concentration of Pro.

Figs. 18A–20A demonstrates suppression of DTT-induced aggregation of BSA by Arg, ArgAd and Pro. As it can be seen from Figs. 18B–20B, where the dependences of the hydrodynamic radius (*R*_h_) of the protein aggregates on time are represented, the protective action of the chemical chaperones is connected with the formation of the protein aggregates of lesser size. The values of parameters *k*_agg_ and *t*_0_ calculated from Eq. (5) at various concentrations of the chemical chaperones are given in Figs. 18C–20C. It should be noted that we do not use Eq. (3) for some kinetic curves, because the extended form of this equation (Eq. (5)) gives a better approximation. To illustrate the expedience of using Eq. (5), the curves calculated from Eqs. (3) and (5) were compared (Fig. 19, 75 mM ArgAd). As can be seen, using Eq. (5) allows us to describe the more extended part of the kinetic curve.

To analyze the dependences of *k*_agg_ on the chemical chaperones concentration, we have used the Hill equation (Eq. (20)). Parameters [L]_0.5_ and *h* calculated from this equation are given in Table 2. The Table also contains the values of the coefficient of determination (*R* ^2^) characterizing the degree of agreement between the experimental data and calculated values. In the case of Pro, the Hill coefficient is equal to unity. However, for Arg, ArgEE and ArgAd the Hill coefficient exceeds unity (*h* = 1.6, 1.9 and 2.5, respectively), suggesting that there are positive cooperative interactions between the chaperone-binding sites in the target protein molecule [96]. Parameter [L]_0.5_ characterizes the affinity of the chaperone to the target protein. As it can be seen from Table 2, among the chaperones studied ArgEE and ArgAd reveal the highest affinity. As for the duration of the lag-period, the increase in the *t*_0_ value is observed with increasing the chaperone concentration (see insets in Fig. 18C–20C).

**Table 2:** The values of parameters of Eq. (20) for suppression of DTT-induced aggregation of BSA by chemical chaperones (*R* ^2^ is the coefficient of determination).

Ligand	[L]_0.5_ (mM)	*h*	*R* ^2^
Arg	116±6	1.6±0.2	0.9871
ArgEE	53±2	1.9±0.2	0.9596
ArgAd	58±2	2.5±0.2	0.9772
Pro	800±120	1.0±0.1	0.9203

Fig. 19C shows the dependences of the *K*_agg_/*K*_agg,0_ ratio on the ArgAd concentration obtained at BSA concentrations equal to 0.5, 1 and 2 mg/ml. The *K*_agg_/*K*_agg,0_ values corresponding to different BSA concentrations fall on the common curve. This result is consistent with the theoretical considerations.

### Combined Action of α-Crystallin and Chemical Chaperones

**Figure 21:**
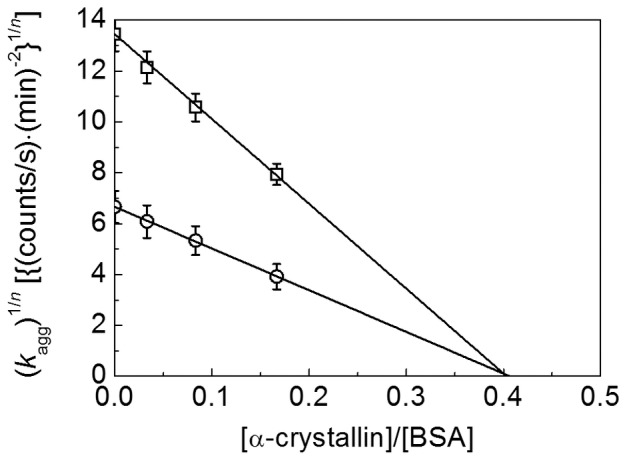
Analysis of combined action of α-crystallin and Arg. The dependences of (*k*_agg_)^1/*n*^ on the [ α -crystallin]/[BSA] ratio in the absence (squares) and in the presence of 100 mM Arg (circles). Conditions: [BSA] = 1 mg/ml, [DTT] = 2 mM, 0.1 M Na-phosphate buffer pH 7.0, 45°C.

In accordance with the principles of analysis of the combined action of protein and chemical chaperones given in the Section “Theory. Quantification of the Chaperone-Like Activity” the following experiments were performed to characterize the mutual inhibitory effects of α-crystallin and Arg. We constructed the (*k*_agg_)^1/*n*^ on the [α-crystallin]/[BSA] ratio plots for BSA aggregation studied in the absence and in the presence of Arg (Fig. 21). The AC_0_ value for α-crystallin was estimated from the initial linear parts of the dependences of (*k*_agg_)^1/*n*^ on the [α-crystallin]/[BSA] ratio. The initial adsorption capacity of α-crystallin in the absence of Arg was found to be 2.48±0.04 BSA monomers per one α-crystallin subunit. The same value of AC_0_ was obtained in the presence of 100 mM Arg (AC_0_ = 2.46±0.02 BSA monomers per one α-crystallin subunit). Thus in the test-system under study α-crystallin and Arg act independently of one another.

When analyzing the combined action of chemical chaperones, parameter *j* (see Eq. (21)) may be used to characterize the interaction between chemical chaperones. As an example we study the combined action of ArgEE and Pro. At [ArgEE] = 50 mM the degree of target protein aggregation inhibition *i*_1_ = 1– *k*_agg_/*k*_agg,0_ was found to be 0.48±0.09. At [Pro] = 800 mM the degree of inhibition (*i*_2_) was 0.42±0.11. The degree of inhibition for the ArgEE (50 mM)+Pro (800 mM) mixture (*i*_1,2_) was 0.73±0.13. Parameter *j* calculated from Eq. (21) was found to be 1.03±0.12. Thus, the action of one chemical chaperone is not dependent on the presence of the other.

## Discussion

The kinetic data obtained in the present work allow us to discuss the mechanism of DTT-induced aggregation of BSA. Taking into account the data on BSA microheterogeneity caused by intramolecular disulfide interchange reactions [64], we can propose the following kinetic scheme of the aggregation process:
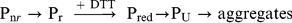
(23)


The first stage is the conformational transition of the initial BSA molecules with sterically hidden disulfide bonds (P_nr_) into the form P_r_ in which disulfide bonds become accessible to the attack by DTT. Next stages are reduction of disulfide bonds (P_red_ is the BSA molecule with reduced disulfide bonds), unfolding of the protein molecule (P_U_ is the unfolded protein) and aggregation of the unfolded protein molecules.

When studying the kinetics of DTT-induced aggregation of α-lactalbumin and insulin, we showed that the initial stage of the aggregation process was the stage of formation of the start aggregates with *R*_h_ of 80–100 nm [18], [84]. No intermediate states between the non-aggregated protein and the start aggregates were detected. This means that the formation of the start aggregates proceeds on the all-or-one principle. Further growth of the protein aggregates occurs as the result of sticking of the start aggregates. The size of the start aggregates is independent of the concentration of the protein involved in aggregation. Thus, the formation of the start aggregates is analogous to the process of micelle formation. In the latter case the micelles of a definite size are formed when the critical monomer concentration is achieved. Such an analogy offers an explanation of why the formation of start aggregates proceeds according to the all-or-none principle. It should be noted that the duration of the lag period (*t*_0_) for the kinetic curves of DTT-induced aggregation of α-lactalbumin tends to decrease with increasing protein concentration and reaches a constant value at rather high concentrations of α-lactalbumin [18].

As in the case DTT-induced aggregation of α-lactalbumin, aggregation of BSA in the presence of DTT is characterized by the decrease in the *t*_0_ value with increasing protein concentration (Fig. 6B). Such a peculiarity of the aggregation kinetics indicates that DTT-induced aggregation of BSA proceeds by a mechanism of nucleation-dependent aggregation [76], [102]–[106]. However, in contrast to α-lactalbumin and insulin, nuclei formed in the course of DTT-induced aggregation of BSA are not capable of assembling into start aggregates. As can be seen in Fig. 5B, there is a monotonous increase in the average value of *R*_h_ of the protein aggregates in the course of aggregation process without separation of non-aggregated and aggregated forms of BSA.

In the present work the rigorous methods for estimation of the anti-aggregation activity of protein and chemical chaperones have been elaborated. When comparing the protective action of protein chaperones, the initial adsorption capacity of the chaperone with respect to the target protein (AC_0_) can be used as a measure of the anti-aggregation activity of chaperones. Taking into account the AC_0_ values for intact and cross-linked α-crystallin (2.50 and 0.212 BSA monomers per one -crystallin subunit, respectively), we can say that cross-linking of α-crystallin results in 11.8-fold decrease in the chaperone-like activity.

It is well known that small heat shock proteins (sHsp) tend to form large oligomers with molecular mass up to 1000 kDa. sHsp oligomers possess high mobility. There are numerous experimental data demonstrating high rate of subunit exchange between oligomers formed by sHsp [6], [107], [108]. The complexes between sHsp and target protein are characterized by high degree of polydispersity [109], [110]. For example, when studying the interaction of Hsp18.1 with firefly luciferase denatured at 42°C by tandem mass spectrometry, Stengel et al. [8] discovered more than 300 Hsp–client protein complexes with different stoichiometry. These complexes are not static entities and can continue to incorporate additional amounts of target protein [109] [8], [110]. Moreover, the Hsp subunits continue to exchange with free sHsp and sHsp–target protein complexes. By contrast, target proteins appear unable to transfer from one complex to another [110].

Since complexation of sHsp with target proteins does not result in the formation of complexes with constant stoichiometry, one may expect that the initial rate of aggregation versus the [sHsp]/[target protein] ratio plot will be non-linear. It has been just such a plot which was obtained for suppression of DTT-induced aggregation of BSA by α-crystallin (Fig. 8). That the dependence of the aggregation initial rate of the [α-crystallin]/[BSA] ratio is of a non-linear character can be interpreted as a decrease in the absorption capacity on α-crystallin with respect to unfolded BSA as the [α-crystallin]/[BSA] ratio increases. It is evident that fixation of the quaternary structure of α-crystallin by cross-linking should yield a linear dependence of initial rate of aggregation on the [α-crystallin]/[BSA] ratio, because in this case monodisperse α-crystallin–target protein complexes are formed. Actually, as can be seen from Fig. 10, the initial rate of aggregation on the [cross-linked α-crystallin]/[BSA] ratio plot is linear. Thus, these data support the idea that non-linear character of the dependence of initial rate of aggregation on sHsp concentration is due to dynamic mobility of quaternary structure of sHsp assemblies and polydispersity of the α-crystallin–target protein complexes.

When comparing the protective action of chemical chaperones, the semi-saturation concentration [L]_0.5_ can be used as a measure of the anti-aggregation activity of chaperones. The lower the [L]_0.5_ value, the higher is the protective power of the chaperone. For example, taking into account the [L]_0.5_ values for Arg and Pro (116 and 800 mM, respectively), we may assert that the protective action of Arg is 7 times higher than that for Pro.

Since chaperones of different classes participate in protein quality control system, we should have the corresponding quantitative methods for estimation of the effects of their combined action at our disposal. The mathematical apparatus described in the Section “Theory. Quantification of the Chaperone-Like Activity” allows us to quantitatively characterize the combined action of the agents possessing the anti-aggregation activity (for example, protein and chemical chaperones).

The data obtained in this study substantiate the use of the test system based on DTT-induced aggregation of BSA for the quantitative estimation of the protective effect of the agents possessing anti-aggregation activity. Parameter *k*_agg_ is used to characterize the initial rate of aggregation measured at different concentrations of the agent under study. The construction of the *k*_agg_ versus the agent concentration plot allows determining parameters, which characterize the protective efficiency of the agent: the adsorption capacity with respect to the target protein (AC_0_) for protein chaperones and the semi-saturation concentration [L]_0.5_ for chemical chaperones. Thus, the test system proposed in the present paper may be used for sampling the agents, which reveal a high protective efficiency and may find application in biotechnological and medical investigations [111].
