# Correction: Quantification of Anti-Aggregation Activity of Chaperones: A Test-System Based on Dithiothreitol-Induced Aggregation of Bovine Serum Albumin

**DOI:** 10.1371/annotation/7c4454a0-209a-4fa0-9fe6-a2e61b80eda2

**Published:** 2014-01-02

**Authors:** Vera A. Borzova, Kira A. Markossian, Dmitriy A. Kara, Natalia A. Chebotareva, Valentina F. Makeeva, Nikolay B. Poliansky, Konstantin O. Muranov, Boris I. Kurganov

The variable "*k*" is incorrectly capitalized at various points throughout the article. In all cases of the use of this variable, except for that in Equation Number 9, this variable should be in lower case. In addition, at multiple points throughout the article, the symbol "α" was not correctly appended to complete the term "α-crystallin."

In addition, in the fifth sentence of the first paragraph of the "Determination of the Initial Rate of Protein Aggression" section in Theory.Quantification of the Chaperone-Like Activity, the variables "*I*", "*t*" and "*A*" are not correctly italicized. In addition, the variable "*t*" is also not correctly italicized in the third sentence of the third paragraph of the same section.

In addition, Equation 10 is incorrect. The correct equation is: *I*=*I*_0_+*k*_agg_(*T*-*T*_0_)^2^,(*T*>*T*_0_). In addition, the first term in Equation 23 is incorrect. The correct term is "P_nr_."

In addition, at multiple points in the "Study of DTT-Induced Aggregation of BSA by Asymmetric Flow Field Flow Fractionation (A4F)" section of the Results, the term "*k*_I_" is incorrectly given as "*k*_l_."

In addition, References 6 and 23 are incorrect. The correct Reference 6 is: Baldwin AJ, Lioe H, Robinson CV, Kay LE, Benesch JL (2011) alphaB-Crystallin

polydispersity is a consequence of unbiased quaternary dynamics.

J Mol Biol 413: 297–309. The correct Reference 23 is: Augusteyn RC (2004) alpha-Crystallin: a review of its structure and function. Clin Exp Optom 87: 356–366.

